# Microbiota-derived butyrate alleviates asthma via inhibiting Tfh13-mediated IgE production

**DOI:** 10.1038/s41392-025-02263-2

**Published:** 2025-06-06

**Authors:** Baichao Yu, Chong Pei, Wenjun Peng, Yongkun Zheng, Ying Fu, Xueqi Wang, Wenjun Wang, Zhiqiang Wang, Yong Chen, Qi Wang, Kameina Zhuma, Yiyuan Gao, Yun Xing, Mengxia Jiao, Ronghua Liu, Feifei Luo, Dan Zhang, Jingbo Qie, Hui Yang, Meiling Jin, Luman Wang, Yiwei Chu

**Affiliations:** 1 Department of Immunology, School of Basic Medical Sciences, Fudan University, Shanghai, China; 2 Department of Pulmonary and Critical Care Medicine, The Third Affiliated Hospital of Anhui Medical University, Hefei, China; 3 Department of Allergy, Zhongshan Hospital, Fudan University, Shanghai, China; 4 Department of Pulmonary and Critical Care Medicine, Shanghai Respiratory Research Institute, Zhongshan Hospital, Fudan University, Shanghai, China; 5 Zhejiang University School of Medicine, Zhejiang University, Hangzhou, China; 6 Shanghai Key Laboratory of Medical Epigenetics and Metabolism, Institutes of Biomedical Sciences, Fudan University, Shanghai, China; 7 Department of Neurology, Children’s Hospital of Fudan University, Shanghai, China; 8 Department of General Surgery, Jiuting Hospital of Songjiang District, Shanghai, China; 9 Shanghai Fifth People’s Hospital, Fudan University, Shanghai, China; 10 Department of Digestive Diseases, Huashan Hospital, Fudan University, Shanghai, China; 11 MOE Innovative Center for New Drug Department of Immune Inflammatory Diseases, Fudan University, Shanghai, China

**Keywords:** Lymphocytes, Respiratory tract diseases

## Abstract

Gut microbiota-derived short-chain fatty acids (SCFAs) impact asthma outcomes, highlighting the importance of understanding the disease mechanisms through the gut–lung axis. In this study, we identified that among SCFAs, butyrate uniquely alleviates asthma through specifically inhibiting a newly identified pathogenic T follicular helper (Tfh) cell subset, Tfh13 cells. Tfh13 cell depletion (*Il13*^*Cre/+*^*Bcl6*^*fl/fl*^) or adoptive transfer of Tfh13 cells in an OVA-induced asthma model conclusively demonstrated their indispensable role in driving anaphylactic IgE production and asthma pathogenesis. Mechanistically, the inhibitory function of butyrate on Tfh13 cells is mediated by the interaction between butyrate and G-protein coupled receptor 43 (GPR43), leading to the suppression of p38 MAPK/NF-κB signaling in Tfh13 cells. To address the clinically observed deficiency of butyrate in patients with asthma and recapitulated in murine models, we developed a novel therapeutic strategy using a butyrate-yielding diet enriched with butylated high amylose maize starch (HAMSB). Remarkably, supplementation with HAMSB diet in murine and humanized asthma models significantly reduced Tfh13 cell frequencies and anaphylactic IgE levels, leading to significantly improved disease outcomes. Our findings not only unveil a novel mechanism underlying butyrate-mediated asthma alleviation, termed the butyrate–Tfh13–IgE axis, but also propose a clinically translatable dietary intervention strategy targeting microbial metabolites for stopping asthma.

## Introduction

As the most extensive microbial community in the human body, the gut microbiota establishes a highly complex and functionally diverse ecosystem within the gastrointestinal tract.^1^ Given its profound influence on host physiology, metabolism, immune function, and nutritional status, the gut microbiota is often regarded as a “metabolic organ“.^2^ The functional interactions between the host and gut microbiota play a pivotal role in maintaining health and contributing to disease pathogenesis. Notably, emerging evidence suggests that gut microbiota influences the homeostasis of distal organs through microbial metabolites.^3–6^ Among these metabolites, short-chain fatty acids (SCFAs) are the best characterized. SCFAs are formed as end-products of dietary fiber fermentation by the gut microbiota, comprising mainly acetate, propionate, and butyrate.^7^ SCFAs have exhibited immunomodulatory effects on multiple intestinal and peripheral immune cells, such as T^8–10^ and B lymphocytes,^11,12^ as well as monocytes.^13^ However, the precise mechanisms by which SCFAs trigger immune cells and their influence on disease outcomes remain not fully understood, representing a critical gap in our understanding of microbiome-host interactions.

Asthma is one of the most prevalent chronic diseases globally, affecting more than 300 million individuals and imposing an ever-increasing substantial burden on healthcare systems worldwide. Allergic asthma is the most common clinical phenotype of asthma, characterized by dysregulated type 2 immune responses and accounting for approximately half to two-thirds of the patients with asthma.^14,15^ Despite the therapeutic efficacy of conventional corticosteroid regimens in symptom management and the emergence of promising monoclonal antibodies targeting type 2 inflammatory mediators, the clinical implementation of these interventions remains constrained. For example, clinical practice shows corticosteroids can cause significant side effects, while biological agents face prohibitive cost barriers, limiting their accessibility and widespread adoption.^16,17^ These persistent therapeutic challenges highlight the urgent need for developing novel, cost-effective, safe, and clinically effective treatment strategies for allergic asthma. Notably, emerging evidence suggests a strong link between decreased levels of SCFAs and elevated allergic asthma prevalence.^18,19^ This growing body of research highlights the exciting prospect that the research focusing on gut microbiota-derived SCFAs may provide novel insights to improve therapeutic effects on allergic asthma. However, the potential immunological mechanisms mediating the relationship between SCFAs and allergic asthma have not been fully elucidated.

Immunoglobulin (Ig)E plays a pivotal role in the pathogenesis of allergic asthma, contributing to the initiation and maintenance of airway inflammation and other asthma-related symptoms.^20^ Traditionally, T helper (Th) 2 cells have been considered the primary drivers of IgE class switching, as demonstrated by studies showing the depletion of interleukin (IL)-4 or GATA Binding Protein (GATA) 3 in Th2 cells compromises type 2 immunity.^21–23^ However, accumulating evidence has challenged the traditional paradigm, convincingly suggesting that the production of IgE is more reliant on follicular T helper (Tfh) cells rather than on Th2 cells.^24–27^ Although Tfh cell-derived IL-4 is crucial for IgE production in both helminth infections and allergic diseases,^27,28^ the IgE generated in allergic diseases exhibits distinct high affinity and exceptionally high anaphylactic capability.^29,30^ This critical functional distinction strongly implies that the precise regulation of IgE production in allergic conditions requires additional Tfh-derived regulatory signaling components beyond IL-4 alone. Notably, Tfh13 cells, a newly identified and functionally specialized Tfh cell subset, have been implicated as a key cell type regulating high-affinity and anaphylactic IgE responses in allergic diseases, including allergic asthma, due to the production of IL-13.^24,31^ Therefore, exploring whether Tfh cells, particularly the Tfh13 cells, mediate the relationships between SCFAs and allergic asthma would be of great significance for elucidating the underlying mechanism and developing new strategies for the prevention and treatment of allergic asthma.

In this study, we discovered that among all SCFAs, butyrate specifically exhibits the strongest association with asthma outcomes. Both patients with asthma and murine asthma models exhibited reduced levels of butyrate and decreased abundance of butyrate-producing bacteria. Notably, among the tested SCFAs, butyrate supplementation demonstrated unique therapeutic efficacy, significantly attenuating asthma symptoms in mice, whereas acetate and propionate showed no comparable effects. Immune profiling conclusively revealed that butyrate selectively inhibits the Tfh13 cell subset. The depletion of Tfh13 cells (*Il13*^*Cre/+*^*Bcl6*^*fl/fl*^) in an OVA-induced asthma model resulted in a significant decrease in IgE^+^ germinal center (GC) B cells and plasma cells, leading to reduced anaphylactic IgE levels. Furthermore, the adoptive transfer of Tfh13 cells further demonstrated their pathogenic role in allergic asthma. Mechanistically, we demonstrated that butyrate engages G-protein coupled receptor 43 (GPR43) to inactivate p38 MAPK/NF-κB signaling, thereby inhibiting Tfh13-mediated IgE production. Based on these findings, we proposed and developed a novel therapeutic strategy using a butyrate-yielding diet enriched with butylated high amylose maize starch (HAMSB). Remarkably, supplementation with the HAMSB diet in murine and humanized asthma models significantly blocked Tfh13 cells and downstream IgE production, eventually efficiently alleviating asthma. Our novel finding highlights the potential of butyrate as a regulator of Tfh13-mediated IgE production, shedding light on its therapeutic implications for allergic asthma.

## Results

### Butyrate plays a key role in mitigating asthma severity

**Fig. 1:**
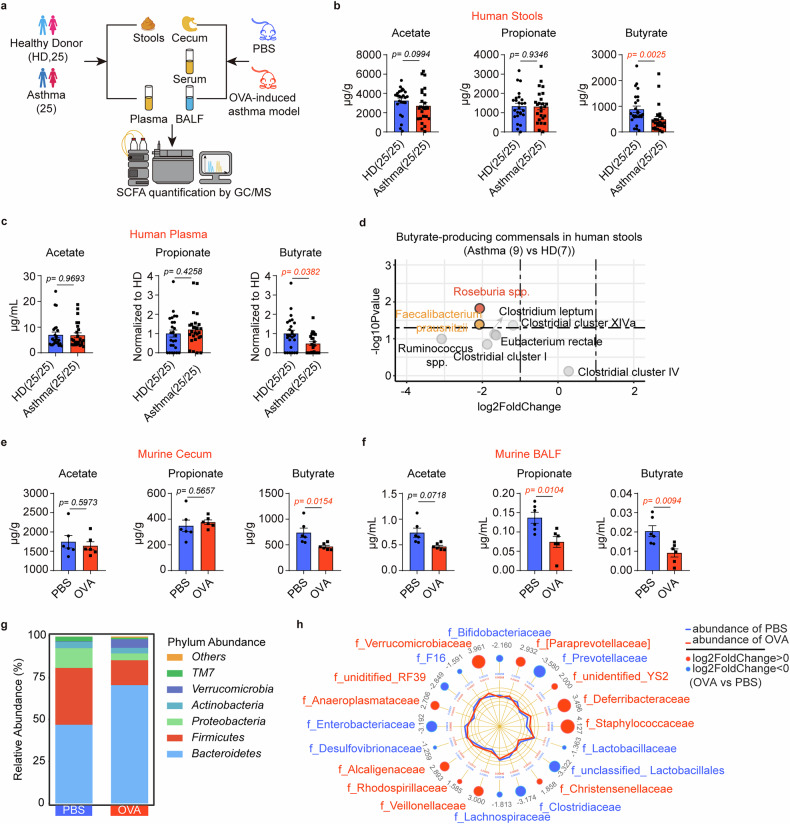
Decreased butyrate levels in patients with asthma and murine models. **a** Experimental scheme for SCFA levels detected by gas chromatography with mass spectrometry (GC/MS) in human and murine samples. **b** Stool acetate, propionate, and butyrate levels in healthy donors (HD, *n* = 25) and patients with asthma (*n* = 25). **c** Plasma acetate, propionate, and butyrate levels in healthy donors (*n* = 25) and patients with asthma (*n* = 25). **d** qPCR for main butyrate-producing bacteria detection in stools of HD (*n* = 7) and patients with asthma (*n* = 9). **e** Cecal acetate, propionate, and butyrate levels in the PBS group and OVA-induced asthma model. (*n* = 6/group) **f** BALF acetate, propionate, and butyrate levels in the PBS group and OVA-induced asthma model. (*n* = 6/group) **g** The taxonomic composition bar plots show the abundance of bacterial communities at the phylum level in the PBS group and the OVA-induced asthma model. (cumulative *n* = 6/group). **h** The radar charts show differences in bacterial taxa at the family level between the PBS group and the OVA-induced asthma model. (cumulative *n* = 6/group). Each symbol represents one mouse. Data combined for at least two independent experiments (**b**, **c**) or were representative of three independent experiments (**e**, **f**). Data represent mean ± SEM analyzed by unpaired *t*-test/nonparametric test (**b**, **c**, **e**, and **f**)

Using gas chromatography-mass spectrometry (GC/MS), we analyzed seven SCFA levels in stool and plasma from patients with asthma/healthy donors, as well as cecal contents, serum and bronchoalveolar lavage fluid (BALF) from OVA-induced murine asthmatic mice/PBS controls (Fig. 1a). In human, butyrate levels were significantly reduced in stool and plasma from patients with asthma, with no inter-group differences observed for other SCFAs (Fig. 1b, c and Supplementary Fig. 1a). We further revealed significantly low abundances of two major butyrate-producing bacteria, *Roseburia spp*., and *Faecalibacterium prausnitzii*, in stool samples from patients with asthma (Fig. 1d). Complementing these human data, cecal, serum and BALF butyrate levels were significantly lower in asthmatic vs control mice while other SCFAs did not differ except propionate in BALF (Figs. 1e, f and Supplementary Fig. 1b, c). Divergent community structures were identified between asthmatic and control mice using 16S rRNA profiling (Fig. 1g), with key butyrate-producing bacteria *Prevotellaceae*, *Clostridiaceae*, and *Lachnospiraceae* significantly depleted in the asthma group (Fig. 1h). Collectively, these results showed decreased butyrate levels along with a reduced abundance of butyrate-producing bacteria in asthma samples.

To determine whether butyrate could mitigate asthma severity compared to other major SCFAs, mice were supplemented with acetate, propionate or butyrate in drinking water starting two weeks prior to disease induction^12^ (Supplementary Fig. 2a). We observed that only butyrate supplementation significantly alleviated asthma severity, characterized by reduced cell counts (total cells and eosinophils) and type 2 cytokines (IL-4, IL-13, and IL-5) in BALF, as well as total and OVA-specific IgG1/IgE levels in serum (Supplementary Fig. 2b–g). Histological analysis also revealed that butyrate supplementation reduced inflammatory cell infiltration and mucus secretion in the airways (Supplementary Fig. 2h, i). In contrast, acetate and propionate supplementation showed no significant effect (Supplementary Fig. 2b–i), indicating the unique and critical role of butyrate in alleviating asthma severity.

To further validate the functional importance of endogenous butyrate, we selectively depleted gram-positive butyrate-producing bacteria using vancomycin, which primarily targets gram-positive anaerobes.^32,33^ Mice received vancomycin in drinking water starting two weeks before OVA induction, with butyrate supplementation via oral gavage beginning one week prior to induction (Supplementary Fig. 4a). To confirm the depletion efficiency, fecal and cecal contents were collected 1 week after vancomycin treatment for 16S rRNA analysis and butyrate detection, respectively (Supplementary Fig. 3a). The findings revealed altered microbiota composition, community structure, species diversity and depleted two major butyrate-producing bacteria, *Ruminococcaceae* and *Lachnospiraceae*, in vancomycin-treated mice compared to those in control mice (Supplementary Fig. 3b–g). Notably, while vancomycin reduced acetate, propionate, and butyrate levels, the butyrate decrease was markedly more pronounced (Supplementary Fig. 3h), providing further evidence of efficient butyrate depletion. As expected, vancomycin-induced butyrate depletion significantly exacerbated asthma pathology, manifesting as increased total cell/eosinophil counts, elevated type 2 cytokines in BALF, heightened OVA-specific IgE levels, and aggravated airway inflammation (Supplementary Fig. 4b–e, g and h–i). While the total IgE/IgG1 and OVA-specific IgG1 levels remained comparable (Supplementary Fig. 4f, g), exogenous butyrate supplementation partially reversed these effects (Supplementary Fig. 4b–i). Notably, type 2 cytokine levels and inflammatory infiltration did not fully recover to those in butyrate-treated asthmatic mice without vancomycin (Supplementary Fig. 4e, i), suggesting that complete rescue may require both exogenous butyrate and an intact microbiota. Collectively, these data highlight the critical role of butyrate in modulating asthma severity by manipulating the gut–lung axis.

### Butyrate constrains IgE production through inhibiting Tfh13 cells

Given the pivotal role of IgE in driving allergic asthma, we sought to elucidate how butyrate inhibits IgE production, as serum from butyrate-supplemented mice failed to elicit anaphylaxis (Supplementary Fig. 5a). We induced IgE production in vitro and assessed whether butyrate directly regulates IgE production (Supplementary Fig. 5b). Surprisingly, the presence of butyrate did not decrease the proportion of IgE^+^ B cells and IgE levels in the supernatant (Supplementary Fig. 5c), indicating that butyrate indirectly regulates IgE production.

**Fig. 2:**
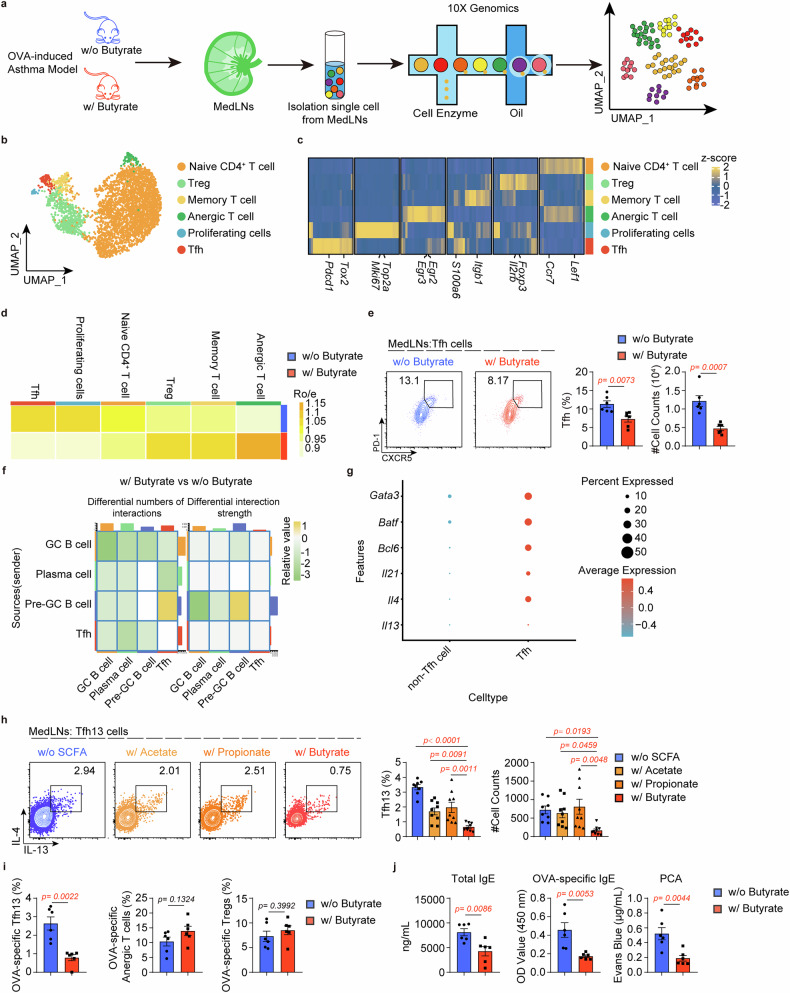
Butyrate inhibits Tfh13 cells to regulate IgE production. **a** Experimental scheme for single-cell RNA sequencing of mediastinal lymph nodes (MedLNs) isolated from asthmatic mice with/without butyrate supplementation. Four mice were pooled per group. **b** Uniform manifold approximation and projection (UMAP) of CD4^+^ T cell clusters. **c** Heatmap of signature genes in each CD4^+^ T cell cluster. **d** The distribution of each CD4^+^ T cell cluster in w/o butyrate and w/ butyrate group. **e** Flow cytometry analysis of the frequency and number of Tfh cells in MedLNs. Representative plots (left) and statistical results (right) were shown. (*n* = 6/group) **f** Cell communication analysis based on CellChat. **g** The expression of selected genes in Tfh and non-Tfh clusters. **h** Flow cytometry analysis of the frequency and number of Tfh13 cells in MedLNs. Representative plots (left) and statistical results (right) were shown. (*n* = 8–9/group) **i** Flow cytometry analysis of the frequency of OVA-specific Tfh13 cells, OVA-specific anergic T cells, and OVA-specific Tregs in MedLNs. Statistical results were shown. (*n* = 6/group) The cells were gated on CD4^+^TCRV2α^+^CD44^+^PD-1^+^CXCR5^+^ OT-II cells. **j** Total and OVA-specific IgE were quantified using ELISA, as well as the anaphylactic capacity of the samples was assessed through PCA assay. (*n* = 6/group) Each symbol represents one mouse. Data combined from three independent experiments (**h**) or were representative of at least two independent experiments (**e**, **i**, and **j**) and represent mean ± SEM analyzed by unpaired *t*-test/nonparametric test (**e**, **i**, and **j**) and one-way ANOVA (**h**)

To investigate the mechanisms by which butyrate modulates IgE production in allergic asthma, we performed single-cell RNA sequencing (scRNA-seq) on mediastinal lymph nodes (MedLNs) isolated from asthmatic mice with or without butyrate supplementation (Fig. 2a). Initial clustering of total cells identified nine distinct subsets encompassing major B and T cell populations (Supplementary Fig. 6a–c). Notably, the proportion and counts of CD4^+^ T cells were reduced with butyrate supplementation (Supplementary Fig. 6c, d). Given this CD4^+^ T cell reduction and their essential role in IgE production, we performed focused reclustering of CD4^+^ subsets, identifying six distinct populations (Fig. 2b, c). Butyrate supplementation reduced the number of Tfh and proliferating cells, while those of regulatory T, memory T, and anergic T cells increased (Fig. 2d). Flow cytometric validation confirmed a significant reduction in the proportion and numbers of Tfh cells within the CD4^+^ T cell subsets, while no significant differences were observed in other CD4^+^ T cell subsets or Tfh viability (Fig. 2e and Supplementary Fig. 6e–i). Further analysis of cell communication showed weaker Tfh-GC B and Tfh-plasma cell interactions (Supplementary Fig. 6j, k and Fig. 2f), indicating that butyrate may constrain IgE production by inhibiting Tfh cells. Notably, the Tfh subset in our scRNA-seq data resembled pathogenic Tfh13 cells co-expressing *Bcl6*, *Gata3*, *Il4*, *Il13*, and *Il21* (Fig. 2g). Importantly, butyrate supplementation significantly reduced expression of IL-13, BCL6, and GATA3 in these cells compared to controls (Supplementary Fig. 6l). Consistent with these findings, butyrate treatment led to reduced plasma cell numbers and decreased expression of *Ighg1* and *Ighe* (Supplementary Fig. 7a, b). As expected, flow cytometry analysis validated lower Tfh13 cells with butyrate supplementation (Fig. 2h), along with reduced IgE^+^/IgG1^+^ GC B and plasma cells in the MedLNs (Supplementary Fig. 7c–f). Importantly, butyrate’s suppression of Tfh13 cells was far more pronounced compared to acetate or propionate (Fig. 2h). Similar reductions in IgE^+^ and IgG1^+^ plasma cells were observed in the lung (Supplementary Fig. 7g), and systemic evaluation revealed parallel decreases in Tfh13 cells, IgE^+^/IgG1^+^ GC B cells and plasma cells in the spleen (Supplementary Fig. 7h–j). To further investigate whether butyrate supplementation eliminates the induction of antigen-specific Tfh13 cells, we adoptively transferred OT-II cells into *Tcrα*^−/−^ mice with or without butyrate supplementation by gavage (Supplementary Fig. 7k). Consistently, the proportion of OVA-specific Tfh13 cells failed to increase in the butyrate-supplemented group (Fig. 2i) and the levels of antigen-specific and anaphylactic IgE were significantly reduced in the butyrate-supplemented mice (Fig. 2j). Notably, while OVA-specific Tregs and anergic T cells remained unchanged (Fig. 2i), the asthma phenotype was significantly alleviated (Supplementary Fig. 7l–p). These results indicate that butyrate alleviates asthma by suppressing Tfh13 cell-driven IgE production.

We next investigated whether butyrate affects IgE production through other cellular subsets apart from Tfh13 cells. Neither Th2 cells nor IL-13^+^ innate lymphoid cells (ILCs) showed significant changes with butyrate treatment (Supplementary Fig. 8a–c). Similarly, follicular regulatory T cells (Tfr), known as regulators of IgE responses,^31,34^ was also unaffected by butyrate (Supplementary Fig. 8d), as did dendritic cells (DCs), which are crucial for the early stage of Tfh cell differentiation^35^ (Supplementary Fig. 8e). To further validate the effect of butyrate on the interaction between Tfh13 and B cells, we sorted Tfh and B cells from MedLNs in asthmatic mice and performed coculture assays with or without butyrate treatment (Supplementary Fig. 8f). The results showed that butyrate treatment significantly reduced OVA-specific Tfh13, IgE^+^, and IgG1^+^ B cell populations (Supplementary Fig. 8g, i). This effect was mediated specifically through IL-13 suppression (Supplementary Fig. 8h), as confirmed by IL-13 neutralization experiments that recapitulated the butyrate-induced inhibition of IgE^+^ B cell generation (Supplementary Fig. 8i). In summary, these results confirm that butyrate reduces IgE production via constraining OVA-specific Tfh13 cell interaction with B cells in an IL-13-dependent manner rather than directly regulating IgE-producing B cells.

### Tfh13 cells are indispensable for IgE production

To ascertain the importance of Tfh13 cells for IgE production, we crossed *Il13*^*Cre*^ mice with *Bcl6*^*fl//fl*^ mice to generate *Il13*^*Cre/+*^*Bcl6*^*fl/fl*^ mice exhibiting specific deletion in Tfh13 cells, as described previously^24^ (Supplementary Fig. 9a). Compared to *Bcl6*^*fl/fl*^ controls, these conditional knockout mice displayed normal T cell development (Supplementary Fig. 9b–d), unaltered splenic T and B cell proportions (Supplementary Fig. 9e, f), and preserved T cell viability, proliferation, and general functionality (Supplementary Fig. 9g–i), demonstrating that these conditional knockout mice maintain overall T cell homeostasis.

**Fig. 3:**
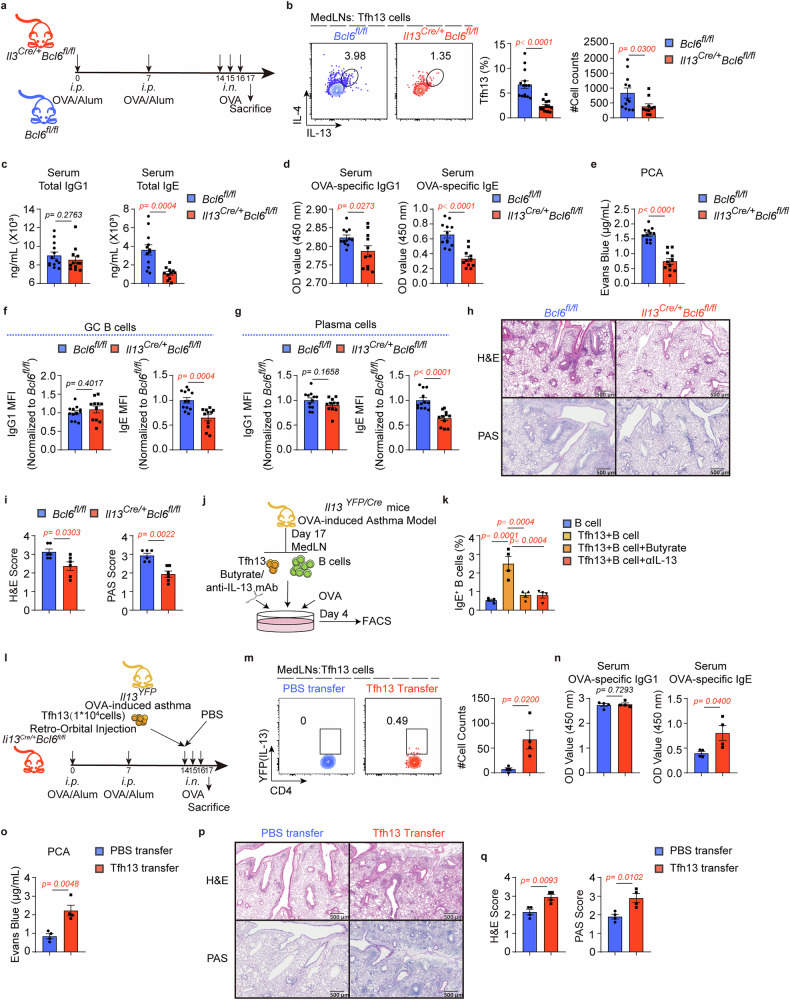
Tfh13 cells are indispensable for IgE production in allergic asthma. **a** OVA-induced asthma models were established in *Bcl6*^*fl/fl*^ and *Il13*^*Cre/+*^*Bcl6*^*fl/fl*^ mice. **b** Flow cytometry analysis of Tfh13 cells in *Bcl6*^*fl/fl*^ and *Il13*^*Cre/+*^*Bcl6*^*fl/fl*^ mice. Representative plots (left) and statistical results (right) were shown. (*n* = 13–14/group) **c** ELISA for total IgG1 and IgE detection. (*n* = 11–13/group) **d** ELISA for OVA-specific IgG1 and IgE detection. (*n* = 11–12/group) **e** Evans blue quantification for anaphylactic IgE detected by PCA assays. (*n* = 11–12/group) **f** Flow cytometry analysis of the MFI of IgG1 and IgE in GC B cells. Statistical results were shown. (*n* = 11–12/group) **g** Flow cytometry analysis of the MFI of IgG1 and IgE in plasma cells. (*n* = 12–13/group) Statistical results were shown. **h** Representative Hematoxylin and Eosin (H&E), as well as Periodic Acid-Schiff (PAS) staining of the lung sections and pathological score were shown in (**i**). Scale bars represent 500 μm. (*n* = 6/group) **j** Experiment scheme. CD4^+^CD44^+^PD-1^+^CXCR5^+^YFP^+^ Tfh13 cells and CD19^+^ B cells were sorted from MedLNs of *Il-13*^*YFP/Cre*^ asthmatic mice and were cocultured in the presence or absence of butyrate. **k** Flow cytometry analysis of IgE^+^ B cells. Statistical results were shown. (*n* = 4/group) About 20 mice were pooled for one sample. Each symbol represents one sample. **l** Experiment scheme. Tfh13 (YFP^+^ Tfh cells) were sorted from MedLNs of IL-13-YFP reporter mice, and 1 × 10^4^ sorted Tfh13 cells pooled from 12–15 IL-13-YFP reporter mice were injected into OVA-immunized *Il13*^*Cre/+*^*Bcl6*^*fl/fl*^ mice via retro-orbital injection, followed by intranasal OVA for three consecutive days. **m** Flow cytometry analysis of YFP^+^ Tfh cells in transferred mice. Representative plots(left) and statistical results(right) were shown. **n** ELISA for OVA-specific IgG1 and IgE detection. **o** Evans blue quantification for anaphylactic IgE was detected by PCA assays. **p** Representative H&E and PAS staining of the lung sections and pathological score were shown in (**q**). Scale bars represent 500 μm. Data combined from at least two independent experiments (**b**–**i**) or were representative of at least two independent experiments (**k**, **l**–**q**) and represent mean ± SEM analyzed by unpaired *t*-test/nonparametric test (**b**–**g**, **i**, **m**–**o**, **q**) and one-way ANOVA (**k**)

We subsequently constructed an OVA-induced asthma model in *Il13*^*Cre/+*^*Bcl6*^*fl/fl*^ and *Bcl6*^*fl/fl*^ mice (Fig. 3a). We observed that *Il13*^*Cre/+*^*Bcl6*^*fl/fl*^ mice exhibited a significant reduction in Tfh13 cells without affecting Tfh cells or IL-13^+^ non-Tfh cells like Th2 cells (Fig. 3b and Supplementary Fig. 9j, k). Notably, although the level of OVA-specific IgG1 reduced marginally, those of total and OVA-specific IgE significantly reduced due to the lack of Tfh13 cells; serum from the *Il13*^*Cre/+*^*Bcl6*^*fl/fl*^ mice also failed to elicit anaphylaxis (Fig. 3c–e). Correspondingly, these mice displayed fewer IgE^+^ GC B cells and plasma cells compared to controls (Fig. 3f, g). Notably, IL-4^+^ Tfh cells remained intact in *Il13*^*Cre/+*^*Bcl6*^*fl/fl*^ mice (Supplementary Fig. 9l), and they may contribute to maintaining a comparable level of total IgG1 and the frequencies of IgG1^+^ GC B and plasma cells (Fig. 3c, f, and g). To further address the crucial role of Tfh13 cells in IgE production, we sorted Tfh13 cells from *Il13*^*YFP/Cre*^ mice after establishing the asthma model and performed in vitro coculture assays with B cells (Fig. 3j). We observed that Tfh13 cells significantly upregulated IgE^+^ B cells, but this effect was reversed in the presence of butyrate or IL-13 neutralization (Fig. 3k). Importantly, butyrate’s suppression of Tfh13-mediated IgE production occurred without affecting cell viability (Supplementary Fig. 9m), excluding cytotoxic effects. Collectively, these results reveal that Tfh13 cells are essential for pathogenic IgE production in allergic asthma.

Furthermore, mice lacking Tfh13 cells exhibited reduced infiltration of inflammatory cells and mucus secretion in the airways following OVA sensitization and challenge compared to *Bcl6*^*fl/fl*^ mice (Fig. 3h, i). To further elucidate the pathogenesis of Tfh13 cells in allergic asthma, we adoptively transferred Tfh13 cells to *Il13*^*Cre/+*^*Bcl6*^*fl/fl*^ mice via retro-orbital injection (Fig. 3l, m). Recipient mice developed elevated OVA-specific and anaphylactic IgE levels (Fig. 3n, o), along with exacerbated pulmonary inflammation characterized by inflammatory cell infiltration into the airway/vessel walls and mucus secretion (Fig. 3p, q). Taken together, these results demonstrate that Tfh13 cells regulate anaphylactic IgE response and contribute to the pathogenesis of allergic asthma.

### Butyrate inhibits Tfh13 function in a GPR43-dependent manner

**Fig. 4:**
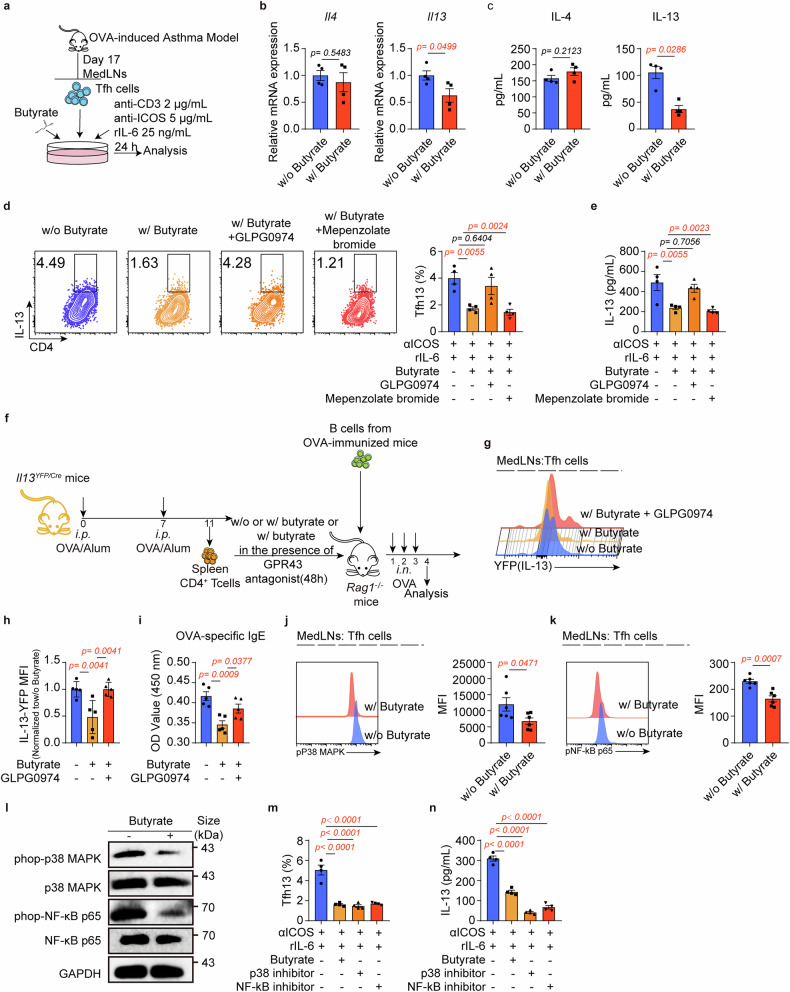
Butyrate inhibits Tfh13 function in a GPR43-dependent manner. **a** Experimental scheme. Tfh cells were sorted from MedLNs from the asthma model and cultured in vitro with mrIL-6, anti-mouse ICOS mAb, and anti-mouse CD3 mAb in the presence or absence of butyrate. Ten to twelve mice were pooled for one sample. Each symbol represents one sample. **b** qPCR for the expression of *Il4* and *Il13*mRNA levels. (*n* = 4/group) **c** IL-4 and IL-13 levels in the supernatant were detected by ELISA. (*n* = 4/group) **d**, **e** Tfh cells were cultured with butyrate in the presence of GLPG094 (GPR43 antagonist) or Mepenzolate bromide (GPR109a antagonist). **d** Flow cytometry analysis of Tfh13 cells. Representative plots (left) and statistical results (right) were shown. (*n* = 4/group) **e** ELISA for IL-13 levels in cultured supernatant. (*n* = 4/group) **f** Experimental scheme. OVA-immunized CD4^+^ T cells from the spleens of *Il13*^*YFP/Cre*^ mice were cultured in vitro in the presence of butyrate with or without GLPG0974 for 48 h. These cells and OVA-immunized B cells were then adoptively transferred to *Rag1*^−/−^ mice, followed by intranasal OVA for 3 days in a row. **g**, **h** Flow cytometry analysis of the MFI of IL-13 in Tfh cells. Representative plots (**g**) and statistical results (**h**) were shown. (*n* = 5/group) **i** OVA-specific IgE levels were detected by ELISA. (*n* = 5/group) **j**, **k** Flow cytometry analysis of phospho-p38 MAPK (**j**) and phospho-NF-κB p65 (**k**) levels in Tfh cells of asthmatic mice with or without butyrate supplementation. Representative plots (left) and statistical results (right) were shown. **l** CD4^+^ T cells from MedLNs of asthma models were cultured in vitro with mrIL-6, anti-mouse ICOS mAb, and anti-mouse CD3 mAb with or without butyrate. Cells were collected at 24 h for p38 MAPK/NF-κB signaling detection via immunoblot. **m** Flow cytometry analysis of Tfh13 cells. Statistical results were shown. (*n* = 4/group) **n** IL-13 levels in the supernatant were detected by ELISA in the presence of p38 MAPK or NF-κB inhibitors. (*n* = 4/group) A representative of three independent experiments was shown. Data represent mean ± SEM analyzed by unpaired *t*-test/nonparametric test (**b**, **c**, **j**, and **k**) and one-way ANOVA (**d**, **e**, **h**–**i**, **m**, and **n**)

To explore how butyrate affects Tfh13 cells, we utilized an in vivo model to induce Tfh13 cells and the sorted Tfh cells for subsequent experiments (Fig. 4a). Butyrate treatment efficiently reduced Tfh cell-derived IL-13 at mRNA and protein levels without impacting IL-4 (Fig. 4b, c), indicating selective IL-13 modulation. Given butyrate’s known actions through G protein-coupled receptors (GPRs; GPR41, GPR43, or GPR109a),^36^ Tfh cells were sorted and incubated with the pan-GPR-signaling inhibitor, pertussis toxin (PTX), in the presence of butyrate. We observed that the inhibitory effect of butyrate on Tfh13 cells was compromised by PTX addition (Supplementary Fig. 10a, b), suggesting that butyrate may inhibit Tfh13 function via GPRs. Gene profiling showed that Tfh cells mainly expressed *Ffar2* (encoding GPR43) and *Hcar2* (encoding GPR109a), unaffected by butyrate (Supplementary Fig. 10c). Therefore, we investigated whether butyrate affected IL-13 production via GPR43 and GPR109a. We cultured Tfh cells with or without the GPR43 antagonist (GLPG0974) or GPR109a antagonist (Mepenzolate bromide) in the presence of butyrate. The decrease in IL-13^+^ Tfh cells and IL-13 levels was compromised by the addition of GLPG0974 but not by adding Mepenzolate bromide (Fig. 4d, e and Supplementary Fig. 10d), suggesting that butyrate exerts its function through GPR43. This finding was further confirmed through adoptive transfer experiments (Fig. 4f), where GLPG0974 treatment restored YFP^+^ (IL-13^+^) Tfh cell numbers and OVA-specific IgE (Fig. 4g–i). Additionally, butyrate can also function as a histone deacetylase (HDAC) inhibitor.^36^ However, although treatment with the HDAC inhibitor, trichostatin A (TSA), significantly reduced the frequency of Tfh13 cells and repressed IL-13 production like butyrate (Supplementary Fig. 10e, f), the inhibitor of HDAC inhibition, Mithramycin A,^37,38^ did not reverse the inhibitory effect of butyrate on Tfh13 cells and IL-13 production (Supplementary Fig. 10g, h), suggesting the effect of butyrate on Tfh13 function is independent of HDAC inhibition.

Activation of the p38 MAPK/NF-κB pathways has been shown to be closely linked to IL-13 production in various immune cells, including T cells.^39–42^ Building upon evidence that SCFAs can suppress inflammation through GPR43-mediated NF-κB inhibition,^43,44^ we investigated whether butyrate regulates IL-13 production in Tfh13 cells via GPR43-mediated inhibition of p38 MAPK/NF-κB activation. As anticipated, our in vivo and in vitro findings demonstrate that butyrate can significantly inhibit phosphorylation of p38 MAPK and NF-κB p65 (Fig. 4j–l). Moreover, the inhibition of these pathways can markedly suppress Tfh13 cells and IL-13 production (Fig. 4m, n). Butyrate also inhibited Tfh cell proliferation, as evidenced by reduced KI67 expression (Supplementary Fig. 10i). Importantly, similar to p38 MAPK/NF-κB inhibitors, butyrate selectively suppressed Tfh13 transcription factors (GATA3 and BCL6) without affecting other cytokines. This butyrate-mediated suppression was GPR43-dependent, as demonstrated by complete reversal with GLPG0974 (Supplementary Fig. 10j, k). Collectively, these results confirm that butyrate targets the GPR43/p38 MAPK/NF-κB p65 axis to inhibit Tfh13 functionality.

### Butyrate-yielding diet alleviates allergic asthma by inhibiting Tfh13-mediated IgE production

**Fig. 5:**
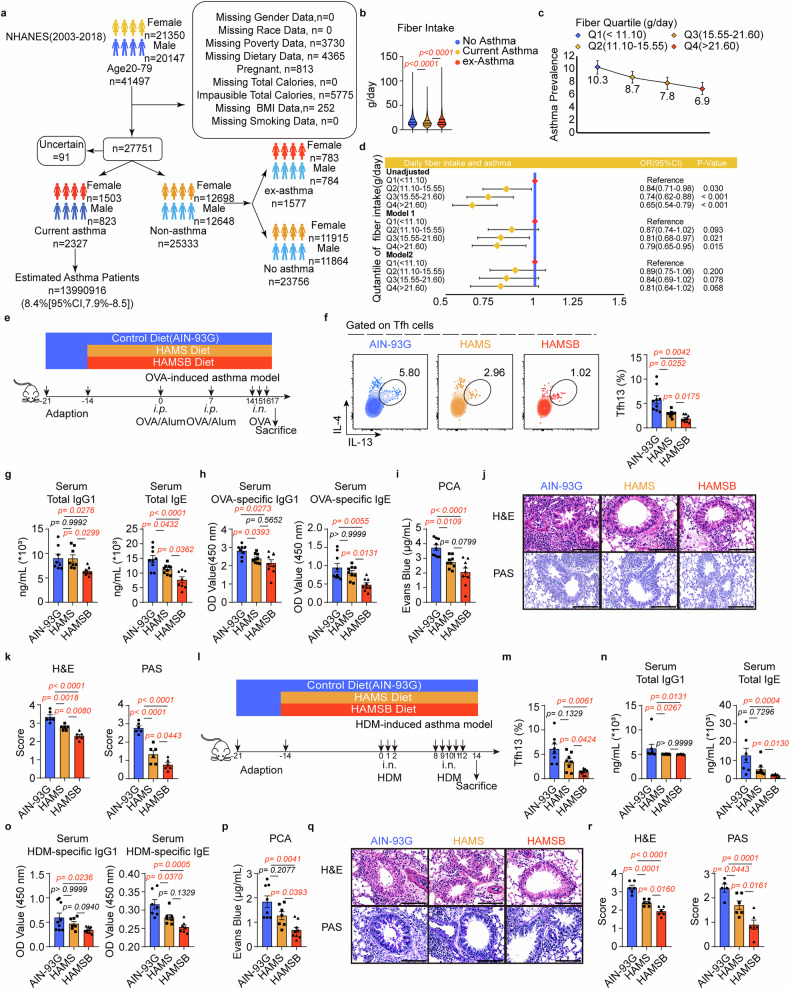
Butyrate-yielding diet protects against allergic asthma. **a** Experimental scheme. The adults (aged 20–79-years-old) who participated in the NHANES survey cycles of 2003 to 2018 were included in our study. Based on the inclusion criteria as shown in (**a**), 27660 subjects were included in our study and divided into three groups: current asthma (2327), ex-asthma (1577), and no asthma (23756). **b**–**d** Dietary fiber intake data were acquired for further analysis. **b** Statistical results of dietary fiber intake in three groups were shown. **c** Dietary fiber intake levels were then divided into four quartiles. The correlation between the four quartiles and asthma prevalence was shown. **d** Multivariable logistic regression model analysis for the correlation between dietary fiber and asthma. Crude, unadjusted by covariates; Model 1, adjusted by age, sex, and race; Model 2, adjusted by age, sex, race, education, poverty index, body mass index (BMI), smoking status, and total calories. **e** Experimental scheme. Three weeks prior to OVA-induced asthma, AIN-93G were supplemented for one week of adaptation and then changed to HAMS or HAMSB diet for two weeks, and throughout the duration of the experiments. **f** Flow cytometry analysis of Tfh13 cells in MedLNs. Representative plots (left) and statistical results (right) were shown. (*n* = 9/group) **g** Total IgG1 and IgE levels were detected by ELISA. (*n* = 8–9/group) **h** OVA-specific IgG1 and IgE levels were detected by ELISA. (*n* = 8–9/group) **i** Anaphylactic IgE levels were detected by PCA assays. (*n* = 9/group) **j** Representative H&E and PAS staining of the lung sections and pathological score were shown in (**k**). The scale bar represents 25 μm. (*n* = 6/group) **l** Experimental scheme. Three weeks prior to HDM-induced asthma, AIN-93G were supplemented for one week of adaptation and then changed to HAMS or HAMSB diet for two weeks, and throughout the duration of the experiments. **m** Flow cytometry analysis of Tfh13 cells in MedLNs. Statistical results were shown. (*n* = 8–9/group) **n** Total IgG1 and IgE levels were detected by ELISA. (*n* = 7–9/group) **o** HDM-specific IgG1 and IgE levels were detected by ELISA. (*n* = 7–9/group) **p** Anaphylactic IgE levels were detected by PCA assays. (*n* = 7–9/group) **q** Representative H&E and PAS staining of the lung sections and pathological score were shown in (**r**). The scale bar represents 25 μm. (*n* = 5–6/group) NHANES database analysis based on R packages: survey and gtsummary (**c**, **d**). Each symbol represents one mouse. Data combined from at least two independent experiments (**f**–**k**, **m**–**r**). Data represent mean ± SEM analyzed by nonparametric test (**b**) and one-way ANOVA/nonparametric test (**f**–**i**, **k**, **m**–**p**, and **r**)

Dietary fiber from plant foods consists of indigestible carbohydrates that escape absorption in the small intestine. Upon reaching the colon, fibers are fermented by anaerobes, generating SCFAs as major end-products.^45^ Given the established correlation between fiber intake and elevated butyrate levels,^46–48^ we hypothesized that dietary fiber-induced increase in butyrate levels may protect against asthma. To identify the relationship between dietary fiber intake and asthma prevalence rate, we analyzed data from the National Health and Nutrition Examination Survey (NHANES) database according to predefined inclusion criteria. Among 27,660 individuals categorized into current asthma (*n* = 2327), ex-asthma (*n* = 1577) and no asthma (*n* = 23,756) (Fig. 5a), current asthma consumed less fiber than other groups (Fig. 5b). Dietary fiber intake level was then divided into four quartiles, revealing an inverse association between dietary fiber intake and asthma prevalence (Fig. 5c). Simultaneously, the lowest quartile was set as the reference group, and crude and adjusted models evaluated the relationship between dietary fiber intake and asthma odds. The crude model showed that dietary fiber intake quartiles were negatively associated with the odds of asthma (Fig. 5d). Covariate adjustment for age, sex, and race attenuated the association, though the third and fourth quartiles remained protective. Despite accounting for all confounders, high fiber intake quartiles (Q3, Q4) were positively associated with lower asthma prevalence than that of the lowest quartile (Q1), with a considerable trend of significance (Fig. 5d). Overall, these findings support the hypothesis that increasing dietary fiber intake protects against asthma.

Resistant starch, a prebiotic fiber known to modulate gut microbiota and enhance SCFA production through fermentation, can be acylated to deliver specific SCFA to the colon.^49–52^ To synthesize starch selectively increasing butyrate, HAMS was acylated with butyric anhydride, yielding butyrate-releasing starch (HAMSB; Supplementary Fig. 11a). The Fourier transform infrared spectrum (FITR) spectra confirmed successful acylation of HAMSB, evidenced by carbonyl C=O vibration detected at 1720–1740 cm^−1^ relative to that of HAMS (Supplementary Fig. 11b). Further, according to the substitution degree determined using acid-base titration, we prepared the HAMSB diet by replacing corn starch in the basic AIN-93G diet with butylated starch (Supplementary Fig. 11a). To investigate whether the HAMSB diet can increase butyrate levels in the distal intestine and affect the gut microbiota, GC/MS and 16S rRNA sequencing were performed prior to inducing the asthma model (Supplementary Fig. 11c). The results showed that HAMSB diet significantly increased cecal butyrate levels compared to control diets (AIN-93G and HAMS diet) (Supplementary Fig. 11d). Furthermore, the HAMSB group exhibited different community structure and composition (Supplementary Fig. 11e–g). Notably, *Firmicutes, Clostridia_UCG-014*, and *Dubosiella*, which are closely associated with butyrate production, were significantly enriched in the HAMSB group (Supplementary Fig. 11h). Thus, the HAMSB diet can remarkably increase butyrate levels and transform gut microbiota.

We further investigated whether the HAMSB diet can reduce asthma severity. Mice were adapted to diets for 1 week before inducing asthma using OVA and then switched to the HAMSB diet starting the second week (Fig. 5e). HAMSB diet was found to reduce inflammation to a greater extent than that using the HAMS and control diet, as evidenced by decreased levels of total cells, eosinophils, and type 2 cytokines (Supplementary Fig. 11i, j). HAMSB further lowered Tfh13 frequency than HAMS (Fig. 5f). Simultaneously, the HAMSB group exhibited lower total IgG1/IgE, and OVA-specific IgE levels than those in the HAMS group (Fig. 5g, h). Lower anaphylactic IgE levels were also observed in the HAMSB group (Fig. 5i). Additionally, the HAMSB group also had fewer infiltrating inflammatory cells and goblet cell hyperplasia (Fig. 5j, k) than those in the HAMS group. Collectively, these findings demonstrate that the butyrate-yielding HAMSB diet effectively alleviates OVA-induced asthma by mitigating multiple pathogenic features.

To further mimic clinical conditions, we constructed a house dust mite (HDM)-induced asthma model to investigate whether the HAMSB diet also protects against allergen-induced asthma (Fig. 5l). Consistent with results observed in the OVA-induced asthma, the HAMSB diet effectively mitigated airway inflammation (Supplementary Fig. 11i, k). Tfh13 cells markedly lowered in HAMSB vs HAMS (Fig. 5m). Concurrently, total IgE, and HDM-specific IgG1/IgE levels also significantly decreased (Fig. 5n, o). The anaphylactic IgE levels were also reduced considerably (Fig. 5p). Moreover, the degree of pulmonary infiltration by inflammatory cells and goblet cell hyperplasia showed enhanced resolution in HAMSB-treated animals compared to that in animals receiving HAMS (Fig. 5q, r). These results suggest the HAMSB diet is a promising regimen for allergen-induced asthma.

### The potential therapeutic effect of butyrate on asthma

**Fig. 6:**
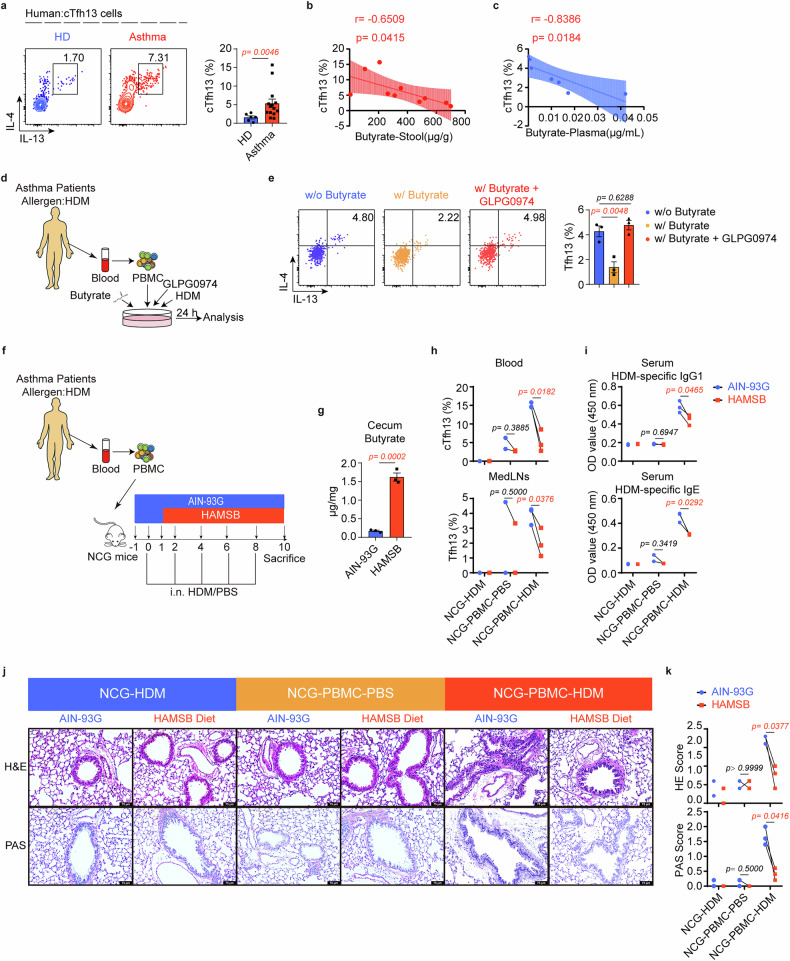
The potential clinical significance of butyrate on asthma. **a** Flow cytometry analysis of cTfh13 cells in human peripheral blood (PB). Representative plots (left) and statistical results (right) were shown. **b**, **c** The correlation between cTfh13 cells and butyrate levels in stools (**b**) and plasma (**c**). **d** Experiment scheme. PBMCs from HDM-allergic patients with asthma were collected and in vitro cultured with HDM, anti-human CD3 mAb, and anti-human CD28 mAb in the presence or absence of butyrate/GLPG0974 for 24 h. **e** Flow cytometry analysis of the frequency of human Tfh13 cells. Representative plots (left) and statistical results (right) were shown. (*n* = 3/group) **f** Experimental scheme. 5 × 10^6^ PBMCs from HDM-allergic patients with asthma or PBS were adoptively transferred to NCG mice. Mice received intranasal HDM (50 μg) or PBS on days 0, 2, 4, 6, and 8. The HAMSB diet was administered from days 1 to 10, starting 1 day after PBMC transfer and during HDM or PBS challenges. Four vials of PBMC from four different donors were pooled for one sample **g** Butyrate levels in the cecum were detected by GC/MS. (*n* = 3/group) **h** Flow cytometry analysis of Tfh13 cells in MedLNs and PB. Statistical results were shown. (*n* = 2–3/group) **i** HDM-specific IgG1 and IgE levels were determined by ELISA. (*n* = 2–3/group) **j** Representative H&E and PAS staining of the lung sections and pathological score were shown in (**k**). The scale bar represents 75 μm. (*n* = 2–3/group) Data were representative of three independent experiments (**e**) and represent mean ± SEM analyzed by unpaired *t*-test/nonparametric test (**a**, **g**), one-way ANOVA (**e**), paired *t*-test (**h**, **i**, and **k**), and Spearman correlation (**b**, **c**)

We subsequently investigated whether Tfh13 cells exist in humans by detecting circulating Tfh13 (cTfh13) cells in healthy donors and patients with asthma. cTfh13 cells increased significantly in patients with asthma vs healthy donors (Fig. 6a). Notably, cTfh13 cell abundance showed a strong inverse correlation with both fecal and plasma butyrate levels in these patients (Fig. 6b, c), implying that butyrate may regulate Tfh13 cells in humans. Building upon these clinical observations, we confirmed GPR43 expression on cTfh cells from both healthy and asthmatic individuals (Supplementary Fig. 12a). Subsequent in vitro stimulation of patient-derived PBMCs demonstrated that butyrate treatment significantly reduced Tfh13 cell proportions, an effect that was completely abrogated by the GPR43 antagonist GLPG0974 (Fig. 6d, e), thereby supporting the role of GPR43 in mediating butyrate’s suppressive effects on human Tfh13 cells.

To further investigate the therapeutic effects of butyrate on asthma, we orally supplemented butyrate in mice for 1 week starting at the challenge phase of the OVA-induced asthma model (Supplementary Fig. 12b). We observed a significant reduction in eosinophil counts (Supplementary Fig. 12c). Strikingly, butyrate supplementation also reduced the proportion of Tfh13 cells (Supplementary Fig. 12d), as well as OVA-specific IgG1 and IgE levels (Supplementary Fig. 12e). Moreover, the ability to induce anaphylactic reaction by serum from the butyrate-supplemented group was weakened (Supplementary Fig. 12f); histological analysis revealed reduced inflammatory cell infiltration and mucus secretion in the airways (Supplementary Fig. 12g, h). To mimic clinical situations, we also examined the therapeutic effects of butyrate on allergen-induced asthma (Supplementary Fig. 12i). Consistent with inhibition in the OVA-induced asthma model, the eosinophil counts and the frequency of Tfh13 cells markedly decreased in the butyrate group (Supplementary Fig. 12j, k), while HDM-specific IgG1 and IgE levels, along with anaphylactic IgE levels, notably decreased (Supplementary Fig. 12l, m). Additionally, pulmonary inflammatory cell infiltration and mucus secretion were significantly reduced after butyrate supplementation (Supplementary Fig. 12n, o).

Further validations were performed using a humanized asthma mouse model with the HAMSB diet (Fig. 6f). Following HDM challenge in PBMC-transferred mice, we observed that the HAMSB diet could substantially diminish the proportion of Tfh13 cells within MedLNs, as well as circulating Tfh13 populations in peripheral blood via increasing butyrate levels (Fig. 6g, h). Concurrently, allergen-specific IgG1 and IgE levels were also markedly reduced, along with significantly decreased pulmonary infiltration by inflammatory cells (Fig. 6i–k). In summary, these data indicate that butyrate exerts therapeutic effects on asthma by inhibiting Tfh13-driven IgE, demonstrating potential clinical applicability via the HAMSB diet.

## Discussion

Accumulating evidence has shed light on the potential protective role of gut microbial metabolites, particularly SCFAs, in asthma.^18,53,54^ Therefore, it is crucial to elucidate the underlying mechanisms. In this study, we provide novel mechanistic insights linking the gut microbiota, its metabolite butyrate, Tfh13 cells, and IgE modulation in asthma pathogenesis. The study findings demonstrate that butyrate acts on Tfh13 cells to constrain IgE production, and butyrate supplementation or a butyrate-yielding diet alleviates asthma in murine and humanized models. Our findings identify butyrate as a promising therapeutic and preventive approach for future asthma management by targeting the gut microbiota-butyrate axis. Overall, this work enhances our understanding of asthma pathogenesis and paves the way for microbiota-directed interventions.

Growing evidence highlights the bidirectional interaction between the gut and lung via the “gut–lung axis” in health and disease settings.^55^ SCFAs, particularly butyrate, are key mediators of this interaction. Our findings reveal a notable decrease in butyrate levels in both human and murine asthma samples. Previous studies have shown that factors such as antibiotic overuse, insufficient dietary fiber intake, and non-breastfeeding are significant contributors to dysbiosis and reduced butyrate levels.^18,53,56,57^ Additionally, systemic inflammatory responses triggered by pulmonary diseases may also disrupt gut microbiota through the gut–lung axis, further decreasing butyrate levels. For instance, pulmonary viral infections in mice have been shown to disrupt gut microbiota homeostasis and decrease SCFA levels by migrating inflammatory cytokines to the gut.^58–60^ Additionally, the respiratory and gastrointestinal systems are part of the common mucosal immune system (CMIS), where lymphocytes can migrate and interact across mucosal tissues.^61^ Antigen stimulation via nasal administration upregulates α4β7 and CCR9 expression on T cells through lung DCs, facilitating their migration to the gut under the influence of Madcam-1 and CCL25.^62^ This suggests the potential migration of pathogenic T cells to the gut in asthma. Therefore, we hypothesize that the observed reduction in butyrate levels in our murine model may result from allergen-induced immune responses, where immune cells and inflammatory cytokines migrate to the gut, disrupting microbiota homeostasis and leading to decreased butyrate production. In humans, we also observed gut microbiota dysbiosis and reduced butyrate levels, though the exact mechanisms are unclear. Whether these changes are driven by asthma, diet, or other environmental factors requires further investigation. Future clinical studies are needed to clarify these causal relationships. In addition, we observed decreased propionate levels in murine BALF, suggesting its potential involvement in asthma pathogenesis. Although previous studies have reported that propionate can alleviate asthma,^63^ our current findings demonstrate that among the three major SCFAs, only butyrate exhibited significant alleviating effects on asthma symptoms, while acetate and propionate showed no such effects in our experimental system. This highlights the unique and critical role of butyrate in asthma modulation, which appears to be distinct from propionate’s reported anti-inflammatory properties.

While direct supplementation with sodium butyrate shows limited bioavailability,^64^ resistant starch enables targeted butyrate production in the distal colon through fermentation with gut microbiota on chemical modification. Butylated resistant starch is known to improve polycystic ovary syndrome,^50^ diabetes,^65^ and hypertension^66^ in murine models and humans. However, its effects on asthma remain unknown. Moreover, our analysis of NHANES data revealed an inverse association between higher fiber intake and asthma prevalence. Based on these backgrounds, we proposed the HAMSB diet to alleviate asthma. As anticipated, HAMSB effectively suppressed Tfh13-mediated IgE generation to prevent asthma via increasing butyrate levels in murine models. Using humanized mice further validated these findings, mirroring its potential efficacy in human asthma. Although clinical HAMSB application requires extensive trials, our findings illustrate the potential of HAMSB for asthma prevention and management. Furthermore, the positive correlation between the number of human cTfh13 cells and allergen-specific IgE levels in other allergic conditions, including food allergy,^24,67,68^ supports our hypothesis that butyrate supplementation or the HAMSB diet could ameliorate allergic diseases by targeting the Tfh13–IgE axis. Despite requiring extensive experimental validation, these results provide an immunological and physiological foundation supporting future investigations into allergic disease pathogenesis and therapies.

A newly identified Tfh cell subset, termed Tfh13 cell, plays a crucial role in driving high-affinity and anaphylactic IgE production in allergic diseases.^24^ While IL-4 is necessary for IgE induction, previous research has demonstrated that IL-13 effectively complements this role by robustly inducing high-affinity IgE in allergic conditions.^24^ IgE production has been shown to rely heavily on IL-13 in allergic diseases.^31,69–72^ In our study, the importance of Tfh13 cells in regulating IL-13-dependent IgE response was further validated through Tfh13 cell-conditional knockout mice and Tfh13 cell adoptive transfer experiments, which collectively confirmed that Tfh13 cells are indispensable for IgE production. Moreover, the knockout and transfer assays directly demonstrated the pathogenic contribution of Tfh13 cells in the context of allergic asthma.

However, the question of whether and how butyrate may regulate this Tfh13-mediated, IL-13-driven IgE response remained unresolved. Our study observed that butyrate selectively diminished Tfh13 frequencies in the MedLNs, without impacting Th2 cells. Importantly, we also found that butyrate primarily affects IL-13 production from Tfh cells. This suggests that butyrate modulates IgE generation specifically by suppressing the IL-13 derived from the pathogenic Tfh13 subset. Further supporting this, Tfh/Tfh13-B cell co-culture experiments showed that butyrate treatment significantly reduced both Tfh13 cells and IgE^+^ B cell populations. Taken together, these findings indicate butyrate’s novel immunoregulatory role in asthma through targeted suppression of the Tfh13–IL-13–IgE axis. This work provides mechanistic insights into butyrate regulation of IgE and highlights Tfh13 cells as a crucial regulatory node modulated by butyrate that is central to IgE responses and pathogenesis in allergic asthma.

Butyrate regulates various immune cell functions through distinct mechanisms, such as acting as an agonist for GPRs or via HDAC inhibition, impacting Treg cells,^8,9^ CD8^+^ T cells,^10^ and NK cells.^73^ However, the mechanism of Tfh cell function regulated by butyrate remains unclear. Butyrate is known to regulate the proliferation and function of Tfr but not Tfh cells via HDAC inhibition in autoimmune arthritis.^74^ Our study provides novel insights into butyrate’s immunomodulatory role in Tfh cells. The results demonstrated that butyrate suppresses IL-13 production in Tfh cells through GPR43 engagement, independent of HDAC inhibition, thereby modulating IgE generation. Importantly, this GPR43-mediated suppression was consistently observed in both murine and human Tfh13 cells, indicating that the conserved butyrate–GPR43–Tfh13 axis represents a promising therapeutic target for allergic asthma. This identifies a distinct mechanism through which butyrate selectively dampens Tfh function in the context of allergic inflammation. IL-13 production, governed by the p38 MAPK/NF-κB signaling axis, is known to impact eosinophils,^42^ mast cells,^41^ and Th2 cells.^40^ Our study reveals that the presence of p38 MAPK and NF-κB inhibitors diminished IL-13 production in Tfh13 cells, underscoring the pivotal role of the p38 MAPK/NF-κB pathways in regulating IL-13 production in Tfh13 cells. Importantly, butyrate specifically targets the GPR43/p38 MAPK/NF-κB axis to selectively suppress IL-13 production and Tfh13-related transcription factors (GATA3 and BCL6) without affecting other cytokines such as IL-4, IL-5, or IL-21. Collectively, our findings differ from those of previous research and unveil the GPR43/p38 MAPK/NF-κB axis as a critical pathway through which butyrate targets the Tfh13–IgE axis in allergic asthma.

**Fig. 7:**
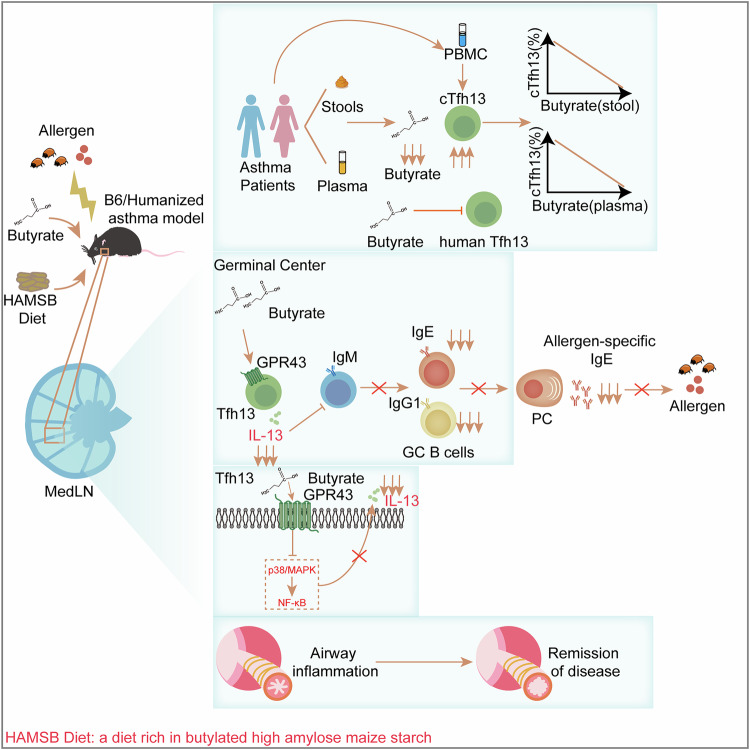
Schematic diagram summarizing the highlights of the study. The study’s key findings are: (1) Low butyrate levels relate to high cTfh13 frequencies in patients with asthma; (2) Butyrate constrains Tfh13-mediated allergen-specific IgE production; (3) Butyrate inhibits Tfh13 function by targeting GPR43/p38 MAPK/NF-κB axis; and (4) The HAMSB diet is a promising preventive and therapeutic approach for asthma. This figure was created using Adobe Illustrator software

In conclusion, our work demonstrates that microbiota-derived butyrate alleviates allergic asthma via inhibiting Tfh13-mediated anaphylactic IgE production in a GPR43-dependent manner; we also propose the potential of butyrate and the HAMSB diet as a preventive and therapeutic approach for asthma (Fig. 7).

## Materials and methods

Reagents, antibodies, and other details and information of experiments are displayed in the Supplementary Materials.

### Mice

Wildtype C57/B6J mice were purchased from Shanghai Jihui Laboratory Animal Co., Ltd. (Shanghai, China). *Il13*^*tml (YFP/cre) Lky*^ mice were purchased from Jackson Laboratories (Bar Harbor, USA) in a Balb/c background. They were backcrossed to a C57BL/6J background. *Bcl6*^*fl/fl*^ mice were kindly provided by Prof. Lilin Ye (Third Military Medical University, China). Tfh13 conditional knockout mice were generated by crossing *Bcl6*^*fl/fl*^ mice with *Il13*^*tml(YFP/cre)Lky*^ mice. *Rag1*^−/−^ and *NOD/ShiLtJGpt-Prkdc*^*em26Cd52*^*Il2rg*^*em26Cd22*^*/Gpt (NCG)* mice were purchased from GemPharmatech Co., Ltd. (Nanjing, China). OT-II mice and *Tcrα*^−/−^ mice were purchased from Cyagen Co., Ltd. (Suzhou, China). In certain experiments, OT-II mice were kindly provided by Prof. Yuanyuan Wei (Fudan University, China). All the mice used were between 5 and 13-weeks-old and were age- and sex-matched for each experiment. They were bred and maintained in the animal facility of Fudan University (Shanghai, China) under specific pathogen-free conditions. All mice used in this study were approved by the Ethics Committee of Animal Care and Use, Department of Laboratory Animal Science, Fudan University.

### Human samples

Human stool and peripheral blood samples were collected from allergic asthma patients and healthy donors with fully informed consent from Hefei First People’s Hospital and Shanghai Zhongshan Hospital. All procedures were approved by the Clinical Ethics Review Board of Hefei First People’s Hospital and Shanghai Zhongshan Hospital. The clinical details were provided in Table S1 and Table S2.

### Mouse models

For the OVA-induced asthma model, mice were injected intraperitoneally with 50 μg OVA (Sigma-Aldrich, USA) in PBS and Alum (InvivoGen, USA) on day 0 and day 7 in a total volume of 100 μL. On days 14–16, mice were anaesthetized by isoflurane (RWD, China) and then treated with OVA (50 μg in PBS) intranasally. On day 17, mice were anaesthetized with 2% pentobarbital sodium, and blood, BALF, lungs, MedLNs, and spleens were collected for further analysis.

For the HDM-induced asthma model, mice were treated with HDM (Greer Labs, USA) intranasally on days 0–2, considered as the sensitization stage. Then, on days 8–12, mice were rechallenged with HDM intranasally. On day 14, mice were anaesthetized with 2% pentobarbital sodium, and blood, BALF, lungs, and MedLNs were collected for further analysis.

### Humanized murine model

PBMCs were obtained from the peripheral blood of asthmatic patients with a known allergen of HDM. 5x10^6^ PBMC were transferred into NCG mice by intravenous injection one day prior to intranasal HDM/PBS. On days 0, 2, 4, 6, and 8, mice were intranasal administered HDM (50 μg) /PBS, followed by feeding the HAMSB diet starting on day 1 and ending on day 10.

### SCFA supplementation

Two weeks prior to the induction of allergic asthma, mice were supplemented with 200 mM sodium acetate, sodium propionate, sodium butyrate (Sigma-Aldrich, USA), or saline as a control in daily water before being exposed to OVA and throughout the duration of the experiment. Alternatively, mice were orally gavaged daily with 1 g/kg body weight of sodium butyrate or saline as a control for 1 week before being exposed to OVA and were throughout the duration of the experiment. In some experiments, butyrate was supplemented in the challenge stage of the asthma model for 1 week.

### Statistical analysis

Statistical analysis was calculated by GraphPad Prism 8.0 and R. Statistical significance was determined by unpaired or paired *t*-test or nonparametric test for comparison of two groups, one-way ANOVA analysis or nonparametric test for comparison of more than three groups, two-way ANOVA or Pearson’s correlation. All results from cytometry assays are presented as the mean ± standard error of mean (SEM). For NHANSE data analysis, Rao-Scott chi-squared test and Wilcoxon rank-sum test were applied to examine the characteristics of the demographic, socioeconomic, and risk factors related to asthma. The association between fiber intake and asthma was evaluated using the multivariable logistic regression models. The multivariable logistic regression models were also adjusted by covariates including age, gender, race, education, poverty, smoking status, calorie intake, and BMI. All the analyses of NHANSE data were performed using the R package survey and gtsummary.

## Supplementary information

Supplementary materials

Uncropped gels for genotyping and immunoblot

## Data Availability

scRNA-seq and 16S rRNA-seq data have been deposited at GEO or SRA, respectively. The accession number for the scRNA-seq database is GSE252579, and the accession numbers for the 16S rRNA-seq database are PRJNA1061240, PRJNA106130, and PRJNA1061315. Any other information required to reanalyze the data reported in this paper is available from the corresponding author, Yiwei Chu (yiweichu@fudan.edu.cn), upon request.
