# Supplementary material for: Microbiota-derived butyrate alleviates asthma via inhibiting Tfh13-mediated IgE production
Source: Signal Transduct Target Ther. 2025 Jun 6;10:181. doi: 10.1038/s41392-025-02263-2 (PMC12141656; doi:10.1038/s41392-025-02263-2)
Supplement: Supplementary file 1 — Supplementary materials [file 41392_2025_2263_MOESM1_ESM.docx]

Supplementary Materials for

**Microbiota-derived butyrate alleviates asthma via inhibiting Tfh13-mediated IgE production**

Baichao Yu^1^, Chong Pei^7^, Wenjun Peng^3,4^, Yongkun Zheng^1^, Ying Fu^1,10^, Xueqi Wang^1^, Wenjun Wang^1^, Zhiqiang Wang^1,8^, Yong Chen^9^, Qi Wang^1^, Kameina Zhuma^1^, Yiyuan Gao^1^, Yun Xing^1^, Mengxia Jiao ^1^, Ronghua Liu^1^, Feifei Luo^6^, Dan Zhang ^1^, Jingbo Qie^1^, Hui Yang^1^, Meiling Jin^3,4*^, Luman Wang^1,2*^, and Yiwei Chu^1,5*^

Correspondence to: yiweichu@fudan.edu.cn; lumanwang@fudan.edu.cn; mljin118@163.com

These authors contributed equally: Baichao Yu, Chong Pei

**The file includes:**

Materials and Methods

Figs. S1 to S12

Tables S1 to S5

References

**Other Supplementary Materials for this manuscript include the following:**

Uncropped gels for genotyping and immunoblots

**Materials and Methods**

**Flow Cytometry and Cell Sorting**

Cells obtained from human samples and murine samples were used for flow cytometry analysis. After centrifugation, the cells were incubated with 1 μg purified anti-CD16/CD32 (Biolegend, USA) to prevent nonspecific binding for 15 min at 4 ˚C. For surface marker staining, cells were incubated with fluorochrome-conjugated antibodies for 30 min at 4˚C. For intracellular cytokine detection, cells were cultured with Cell Stimulation Cocktail (plus protein transport inhibitors) (Invitrogen, USA) for 5 h and were then stained with surface markers, followed by permeabilization and incubation with intracellular antibodies. For intra-nuclear transcription factor staining, after surface staining, cells were then stained with intra-nuclear transcription factor antibodies using the Foxp3-Transcription Factor Staining Buffer Set (Invitrogen, USA). For phosphorylated protein staining, cells were fixed and permeabilized by IC fixation buffer and methanol, respectively, followed by surface marker and intracellular phosphorylated protein staining. For OVA-specific T cell staining, cells were stained with I-Ab OVA_323-339_ Tetramer-APC, followed by surface marker staining. The flow cytometry data were collected using BD FACSCelestaTM Cell Analyzer (BD Biosciences, USA), and were analyzed using FlowJo V10 software (BD Biosciences, USA). For cell sorting, the cells were sorted using MoFlo XDP High-Speed Cell Sorter/ CytoFLEX SRT (Beckman, USA).

**Single cell RNA (scRNA) sequencing**

Total cells were sorted from MedLNs of butyrate-treated or -untreated OVA-induced asthma model. The single cells were obtained by the method of grinding through a 70 μm cell strainer. Single cell capture, barcoding and library preparation were performed using 10x Genomics Chromium™ by NEO Biotech Co., Ltd (Shanghai, China). The CellRanger 6.0.1 was used to convert Illumina base call (bcl) files to FASTQ files. We used the following R packages for the further analysis, including Seurat 4.3.0 and CellChat 1.1.3. We excluded low quality cells according to following criteria: 1) percentage of mitochondrial genes above 10; 2) the number of genes detected per cell was less than 200; 3) doublet cells. Clustering and UMAP analysis were performed following standard Seurat pipeline^1^. Cell subsets were annotated according to the top genes obtained by running the FindMarkers function. Cell communication was analyzed between Tfh cells and B cell subsets using CellChat as described previously^2^.

**NHANES database**

We utilized data from the NHANES to obtain baseline characteristics of our study population. NHANES is an ongoing cross-sectional nationwide survey designed to assess the health and nutritional status of adults and children in the United States. It is a major program of the National Center for Health Statistics (NCHS) part of the U.S. Centers for Disease Control and Prevention (CDC). To improve statistical power for data analysis and to represent the entire US population, study subjects were selected using stratified multistage probability sampling, including oversampling of minority, low-income, elderly subjects, etc^3^. Informed consent was obtained from all participants before enrollment in the study. Complete details of the research methodology and full dataset are publicly accessible on the project website(<http://www.cdc.gov/nchs/nhanes.htm>).

The adults (aged 20-79 years old) who participated in NHANES survey cycles of 2003 to 2018 were included in our study. Only participants with available data for the variables of interest were included in the analytic sample. Briefly, pregnant women and participates with missing gender, race, poverty index ratio, dietary fiber intake, smoking status, body mass index (BMI) and total calorie intake data were excluded. Additionally, implausible calorie intake data (women >6000 or < 600 kcal/d; men >8000 or < 800 kcal/d) were also excluded. Asthma was defined based on responses to interviewer-administered questionnaires conducted by trained interviewers. Current asthma was defined based on affirmative responses to the two questions asked during the interview: "Has a doctor or other health professional ever told you that you have asthma?" and "Do you still have asthma?". Participants who responded "no" to either question were classified as non-asthma. Among them, those who reported ever being diagnosed with asthma but do not currently have it were further classified as ex-asthma, while those who responded "no" to both questions were classified as no asthma. Also, participants whose asthma status could not be determined due to an uncertain response were excluded from the analysis. All populations are described in Table S3.

**Antibiotics treatment**

For the depletion of butyrate-producing bacteria, 2 weeks prior to the induction of asthma, vancomycin (500 mg/L; Meilunbio, China) was utilized in the drinking water and throughout the experiment. After 1 week treatment of vancomycin, mice were oral gavage daily with 1 g/kg body weight of sodium butyrate or saline and throughout the duration of the experiment.

**16S rRNA sequencing**

Murine stools were quickly transferred to -80 ℃ for storage after collection. The 16S rRNA sequencing was performed on Illumina Navaseq6000 platform by OE Biotech Co., Ltd (Shanghai, China). The V3-V4 regions of bacterial 16S rRNA genes were amplified with the primers: 343F-TACGGRAGGCAGCAG and the 798R-AGGGTATCTAATCCT.The data were analyzed on the online platform of OE Cloud platform (<https://cloud.oebiotech.com>). In some experiments, murine cecum contents were collected and quickly transferred to -80 ℃ for storage. The 16S rRNA sequencing was performed on Illumina Nextseq2000 platform by Majorbio Biotech Co., Ltd (Shanghai, China). The data were analyzed on the online platform of Majorbio Cloud platform (<https://cloud.majorbio.com/>).

**Collection and analysis of BALF**

Mice were euthanized and were then irrigated twice with 1 mL PBS to obtain BALF. After centrifugation, the supernatant was obtained and mouse uncoated ELISA kit (Invitrogen, USA) was used to detect IL-5, IL-13 and IL-4. The precipitation was re-suspended with 1mL PBS for cell counting and flow cytometry.

**Cell preparation**

MedLN and spleen cells were harvested post-mortem by the method of grinding through a 70 μm cell strainer (SORFA, China). The erythrocytes in spleen cells were lysed using 1X RBC Lysis Buffer (Invitrogen, USA). The lung single cell preparation was performed as described previously^4^. Briefly, after lavage, the lungs were removed from the mice and chopped into pieces and digested in 2 mL digestive solution (RPMI 1640, 1 mg/ml collagenase IV (Sigma, USA), and 5 U/ml DNase I (Sigma, USA)) for 1 h at 37 ℃. The digested lung tissues were then filtered using a 70 μm cell strainer, and the erythrocytes were lysed using 1X RBC Lysis Buffer (Invitrogen, USA). For the further enrichment of mononuclear cells in the lung, we utilized density gradient centrifugation to separate mononuclear cell by using 40% and 70% Percoll (Cytiva, USA) solution. The enriched cells were washed and prepared for further experiments.

**Passive cutaneous anaphylaxis (PCA) assay**

PCA assay was performed as described previously^5^. Briefly, 20 μL serum from OVA/HDM-immunized mice that received butyrate/HAMSB treatment or not were injected into ear pinnae of naïve C57/B6J recipients. 24 h later, 100 μg OVA/HDM with 1% Evans Blue were injected intravenously into the recipient mice. 30 min later, the mice were euthanized and ear pinnae were harvested and incubated in formamide for 48 h at 56 °C to release the Evans Blue. The degree of vascular leakage was detected by Infinite® M200 PRO reader (TECAN, USA) at 620 nm and Evans Blue was used as the standard.

**Histopathology assessment**

The irrigated lungs from each mouse were collected and fixed in 4% paraformaldehyde. The lungs were then paraffin-embedded, sliced, and stained with Haemotoxylin and Eosin(H&E) or Periodic acid-Schiff (PAS). The stained sections were evaluated microscopically by the Leica DFC7000T microscope. The degree of lung inflammation and goblet cell hyperplasia was scored on a scale of 0-4 as previously described^6^. Briefly, lung inflammation was graded as follows: 0: normal; 1: mild inflammation; 2: moderate inflammation; 3: severe inflammation; 4: very severe inflammation. For goblet cell hyperplasia, five-point grading system was used: 0, no PAS-positive cells; 1, <25% PAS-positive cells; 2, 25%-50% PAS-positive cells; 3, 50-75% PAS positive cells; 4, > 75% PAS-positive cells. Five fields were randomly selected for scoring.

**Immunofluorescence**

Paraffin sections of murine MedLNs were stained with anti-GL-7 (Invitrogen, USA), anti-CD4 (Abcam, USA), anti-Rat HRP (Invitrogen, USA) and DAPI, followed the manual of 4-color Fluorescence kit (Panovue, China). The stained sections were evaluated microscopically by the Leica DFC7000T microscope.

**Enzyme linked immunosorbent assay (ELISA)**

For serum total antibodies, mouse IgE or IgG1 uncoated ELISA kit (Invitrogen, USA) were used for the detection of total IgE or IgG1 according to manufacturer's recommended protocol.

For serum antigen-specific antibodies, 20 μg/mL OVA or HDM in PBS was coated on 96-well Costar® Assay plate (Corning, USA) overnight at 4 °C. Plate was blocked with 5% bovine serum albumin (ABcone, China) in PBS for 2 h at room temperature and then diluted serum was added into the plate and incubated for 2 h at room temperature. Anti-IgE-HRP or anti-IgG1-HRP (Southern Biotech, USA) was used for the detection of antigen-specific IgE or IgG1, respectively for 90 min at room temperature. Plates were developed with TMB substrate solution for 10-15 min and stopped with dilute sulphuric acid before reading at 450 nm on Infinite® M200 PRO reader.

For BALF and cultured supernatant cytokine detection, mouse IL-4, IL-5, IL-13 or IL-21 uncoated ELISA kit (Invitrogen, USA) were used for the detection of IL-4, IL-5, IL-13 or IL-21 according to manufacturer's recommended protocol.

**Tfh cells *in vitro* culture**

Tfh (CD4^+^CD44^+^CXCR5^+^PD-1^+^) cells were sorted from MedLNs of OVA-induced asthma model using fluorescence activated cell sorting (FACS). Twenty-five thousand cells/well were cultured in 96-well U-bottom plates in IMDM containing 10% fetal bovine serum (FBS), 1% penicillin-streptomycin solution with 25 ng/mL mouse recombinant IL-6 (mrIL-6; Peprotech, USA), 2 μg/mL purified anti-mouse CD3 mAb (Biolegend, USA), 5 μg/mL purified anti-mouse ICOS mAb (Biolegend, USA) with or without 0.4 mM sodium butyrate (Sigma, USA) for 24 h. In certain experiments, 1 μg/mL Pertussis toxin (List Labs, USA), 10 nM GLPG0974(Sigma, USA), 10 nM mepenzolate bromide (MCE, USA), 1 nM Trichostatin A (TSA; MCE, USA), 10 nM Mithramycin A (MCE, USA), 40 μM p38 MAPK-IN-1 (MCE, USA) or 10 μM JSH-23 (MCE, USA) were added into cell culture system.

**Real-time reverse transcription PCR analysis**

For cultured cells, Tfh cells were sorted from MedLNs derived from OVA-induced asthma model and cultured in the presence of anti-mouse CD3 mAb, anti-mouse ICOS mAb, and mrIL-6 with or without sodium butyrate for 24 h. Total RNA was extracted by RNAsimple Total RNA Kit (TIANGEN, China) and reverse transcribed into cDNA using Hifair®II 1st Strand cDNA Synthesis SuperMix for qPCR (gDNA digester plus) (YEASEN, China).The real-time PCR was performed on ABI7500 Thermocycler (Applied Biosystems, USA) in 20 μL reaction system containing cDNA, primers, distilled water, and Hieff UNICON® qPCR SYBR Green Master Mix (YEASEN, China). The reaction procedure was set up according to manufacturer's recommended protocol. The 2^-ΔΔCt^ calculation method was used to compute relative gene expression compared to *β-Actin*. Primers for target genes are listed in Table S4.

For human and mouse fecal samples, fecal genomic DNA was extracted using a TIANamp stool DNA Kit (TIANGEN, China) according to the manufacturer’s instructions. The real-time PCR was performed on LightCycler®480 II (Roche, Switzerland) in 10 μL reaction system containing fecal genomic DNA, primers, distilled water, and Hieff UNICON® qPCR SYBR Green Master Mix (YEASEN, China). The reaction procedure was set up according to manufacturer's recommended protocol. The 2^-ΔΔCt^ calculation method was used to compute relative abundance compared to universal 16S rRNA gene. Primers for specific microbiota are listed in Table S4.

**Preparation of butylated starch**

The preparation of butylated starch was performed as previously described^7^. Briefly, a certain amount of high amylose maize starch was weighed and mixed with an appropriate of deionized water to form a 40% (w/v) starch solution. The mixture was then heated to 35-40 ℃ in water bath and stirred continuously throughout the experiment. The pH of starch solution was adjusted to 8-9 with 1 mol/L NaOH. The butyric anhydride (50% of starch mass) was slowly added into the starch solution in 90 min, while using 1 mol/L NaOH to maintain the pH of the reaction system between 8-9. After adding the butyric anhydride, the mixture was stirred continuously until 2 h. The reaction was then terminated by adjusting the pH to 5.5-6 using 1 mol/L HCL. The starch solution settled naturally, and then the supernatant was discarded. The sediment was washed by distilled water for twice, and dried at 55 ℃ overnight. Finally, the dried particles were ground into powder to obtain butylated starch.

**Determination of degree of substitution**

The degree of substitution (DS) of acylated starch was determined by acid-base titration. Butylated starch and normal starch were weighed and reacted with NaOH under alkaline conditions for 90 min. The excess NaOH is then titrated with a standard solution of HCL, and the degree of substitution of the acylated starch was calculated according to the following formula^7^:

$$A=(\frac{V_{2}}{m_{2}}-\frac{V_{1}}{m_{1}})\times\frac{0.5\times M\times100}{1000}$$

$$DS=\frac{162\times A}{100\times M-(M-1)\times A}$$

A: Mass fraction of acyl

V_2_: Volume of HCL standard solution consumed by non-butylated starch, mL

m_2_: Weight of non-butylated starch, mg

V_1_: Volume of HCL standard solution consumed by butylated starch, mL

m_1_: Weight of butylated starch, mg

M: Molar mass of substituent, g/mol (71 for butyryl)

162: Relative molecular mass of maize starch

**Preparation of HAMS and HAMSB diet**

The amount of butylated starch to replace maize starch in the standard diet (per kg) was determined according to the following formula:

$$0.25\times150=DS\times m$$

DS: degree of substitution

m: additive amount of butylated starch

Control starch (150 g/kg) and butylated starch (DS=0.23, 163 g/kg) replaced maize starch of standard diet to prepare the HAMS and HAMSB diet, respectively. AIN-93G diet was considered as standard diet. The diet was processed by Shuyu Biotech Ltd (Shanghai, China).

**Fourier transform infrared spectrum (FTIR) spectra**

FITR spectra of butylated starch and control starch were performed by Nicolet IS5 (Thermo Fisher Scientific Inc, USA). The sample were detected by using attenuated total refraction (ATR) method and wave number ranged between 600-4000 cm^-1^.

**B cells *in vitro* culture**

MedLN B cells (CD19^+^CD4^-^) were sorted from OVA-induced asthma model through FACS. Two hundred thousand cells were cultured in 96-well plates in RPMI-1640 containing 10% fetal bovine serum (FBS), 1% penicillin-streptomycin solution with 10 ng/mL mrIL-4, 50 ng/mL mrL-13, 2 μg/mL anti-mouse CD40 mAb with or without butyrate treatment for 3 days.

**Human PBMC *in vitro* culture**

PBMC were sorted from patients with asthma whose allergen are HDM. Two hundred thousand cells were cultured in 96-well U-bottom plates in IMDM containing 10% fetal bovine serum (FBS), 1% penicillin-streptomycin solution with 40 μg/mL HDM, 2 μg/mL purified anti-human CD3, 2 μg/mL purified anti-human CD28 (Biolegend, USA) with or without 0.4 mM sodium butyrate and 10 nM GLPG0974 (Sigma, USA) for 24 h.

**SCFA analysis by** **GC/MS**

Human samples (stool and plasma) and murine samples (BALF, serum and cecum) were collected after liquid nitrogen frozen and quickly transferred to -80 °C for storage. For stool and cecum samples, take an appropriate amount of sample (about 50 mg) and place it into a 2 mL centrifugal tube. Add 50 μL of 15% phosphoric acid solution, then add 125 μg/mL of internal standard (isocarproic acid) 100 μL solution and 400 μL of diethyl ether. Mix for 1 min, and then centrifuge at 4 °C with 12000 rpm for 10 min. Take the supernatant for subsequent detection and analysis. For plasma, serum and BALF samples, take an appropriate amount of sample (about 100 μL) and place it into a 2 mL centrifugal tube. Add 50 μL of 15% phosphoric acid solution, then add 75 μg/mL of internal standard (isocarproic acid) 10 μL solution and 140 μL of diethyl ether. Mix for 1 min, and then centrifuge at 4 °C with 12000 rpm for 10 min. Take the supernatant for subsequent detection and analysis. All of the GC/MS analysis was performed by Biodeep Biotech Co., Ltd (Suzhou, China).

**Butyrate analysis by GC/MS**

Murine stool or cecal samples were collected after liquid nitrogen frozen and quickly transferred to -80 °C for storage. The tissue was homogenized with 1 mL of 0.5% phosphoric acid solution at 4 °C, then an appropriate amount of 0.5% phosphoric acid solution was added and vortexed for 1 min. Then the same volume of tert-Butyl methyl ether (MTBE, Sigma, USA) as 0.5% phosphoric acid solution was added and vortexed for 1 min. The mixture was centrifuged at 4000 rpm at 4 °C for 10 min. 100 μL of the MTBE layer was transferred to a GC bottle, and then 1 μL of the internal standard (Butyrate-d7 Acid) was added to each sample for further analysis. GC/MS analysis was performed with Agilent 7890A/5975C on the Core Facility of Shanghai Medical College, Fudan University.

**Adoptive transfer**

CD4^+^ T cells were purified from spleens of OVA-immunized IL-13-YFP reporter mice using CD4^+^ T cell isolation kit (Stemcell, Canada). These purified cells were cultured in 48-well plates with 2 μg/mL anti-mouse CD3 mAb (Biolegend, USA), 2 μg/mL purified anti-mouse CD28 mAb (Biolegend, USA) with or without sodium butyrate (Sigma, USA) and GLPG0974(Sigma, USA) for 48 h. The transferred B cells were purified from spleen of OVA-immunized mice using B cell isolation kit (Stemcell, Canada). These B cells (3x10^6^ cells) and T cells (1x10^6^ cells) were respectively intravenous injected into *Rag1*^-/-^ mice, followed by intranasal OVA for three consecutive days.

For OVA-specific Tfh13 detection, OT-II cells were purified from spleens and lymph nodes of OT-II mice using CD4^+^ T cell isolation kit (Stemcell, Canada). 1x 10^7^ OT-II cells were intravenous injected into *Tcrα^-/-^* mice, followed by butyrate by gavege prior one week to the induction of OVA-induced asthma model.

For Tfh13 adoptive transfer, IL-13-YFP^+^ Tfh cells were sorted from MedLNs from IL-13-YFP reporter mice with asthma by using FACS. 1x 10^4^ sorted Tfh13 cells were injected into OVA-immunized *Il13^Cre/+^Bcl6^fl/fl^* mice via retro-orbital injection, followed by intranasal OVA for three consecutive days.

**Coculture *in vitro***

Tfh (CD4^+^CD19^-^CXCR5^+^PD-1^+^) or Tfh13 (CD4^+^CD19^-^CXCR5^+^PD-1^+^YFP^+^ (IL-13)) cells and B cells (CD19^+^CD4^-^) cells were sorted from MedLNs of OVA-induced asthma models. 50 thousand Tfh cells or 20 thousand Tfh13 cells and 150 thousand or 60 thousand B cells were cocultured in 96-well U-bottom plates in IMDM containing 10% fetal bovine serum (FBS), 1% penicillin-streptomycin solution for 4 days with OVA (40 μg/mL) in the presence or absence of sodium butyrate (0.4 mM)/ IL-13 neutralization antibody (10 μg/mL; Invitrogen, USA).

**Immunoblots**

CD4^+^ T cells were purified from MedLNs of asthma models using CD4^+^ T cell isolation kit (Stemcell, Canada). These purified cells were cultured in 48-well plates with 2 μg/mL anti-mouse CD3 mAb (Biolegend, USA), 5 μg/mL purified anti-mouse ICOS mAb (Biolegend, USA) and 25 ng/mL mrIL-6 (Peprotech, USA) with or without sodium butyrate (Sigma, USA) for 24 h. These cells were lysed in RIPA lysis buffer (Epziyme, China) with Protease and Phosphatase Inhibitor Mini Tablets (Thermo Fisher Scientific, USA) on ice for 15 min. The lysed supernatants were collected after centrifugation and then boiled in sample buffer at 100 ℃ for 10 min. The proteins were separated by SDS-polyacrylamide gel electrophoresis and then transferred to a polyvinylidene difluoride membrane (0.45 μm, Millipore, USA) by a transfer apparatus. The membrane was blocked by 5% bovine serum Albumin (ABcone, China) in Tris buffered saline with Tween 20 and incubated with primary antibody including NF-κB p65 mAb (CST, USA), p38MAPK mAb (Abclonal, China), phospho- NF-κB p65 mAb (Invitrogen, USA), phospho-p38MAPK mAb (CST, USA) and GAPDH pAb (YEASEN, China). After incubation of primary antibodies overnight at 4 ℃, the membranes were incubated with Anti-Rabbit IgG, HRP-linked Antibody (CST, USA) and were visualized using a Femto-Sensitive ECL Solution (GeneBrick, China).

**Supplementary Figures**


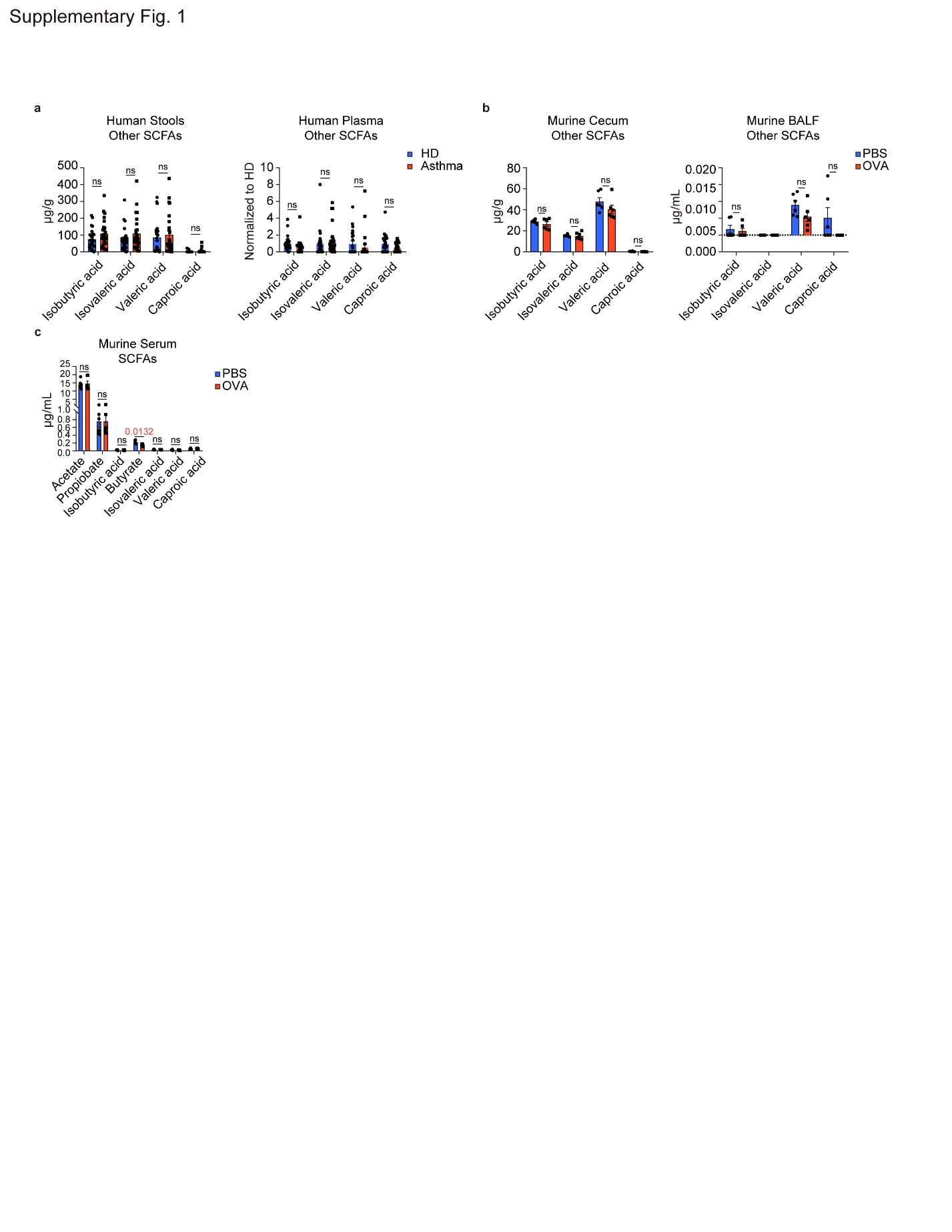


**Supplementary Fig. 1**. Other SCFA levels in human and murine samples. **a** Other SCFA levels in human stool and plasma samples. (n=25/group) **b** Other SCFA levels in murine cecal and BALF samples. (n=6/group) **c** SCFA levels in murine serum samples. (n=6/group). Data combined from at least two independent experiments (**a**) or were representative of three independent experiments (**b, c**). Data represent mean ± SEM and are analyzed by unpaired t test/nonparametric test (**a-c**)


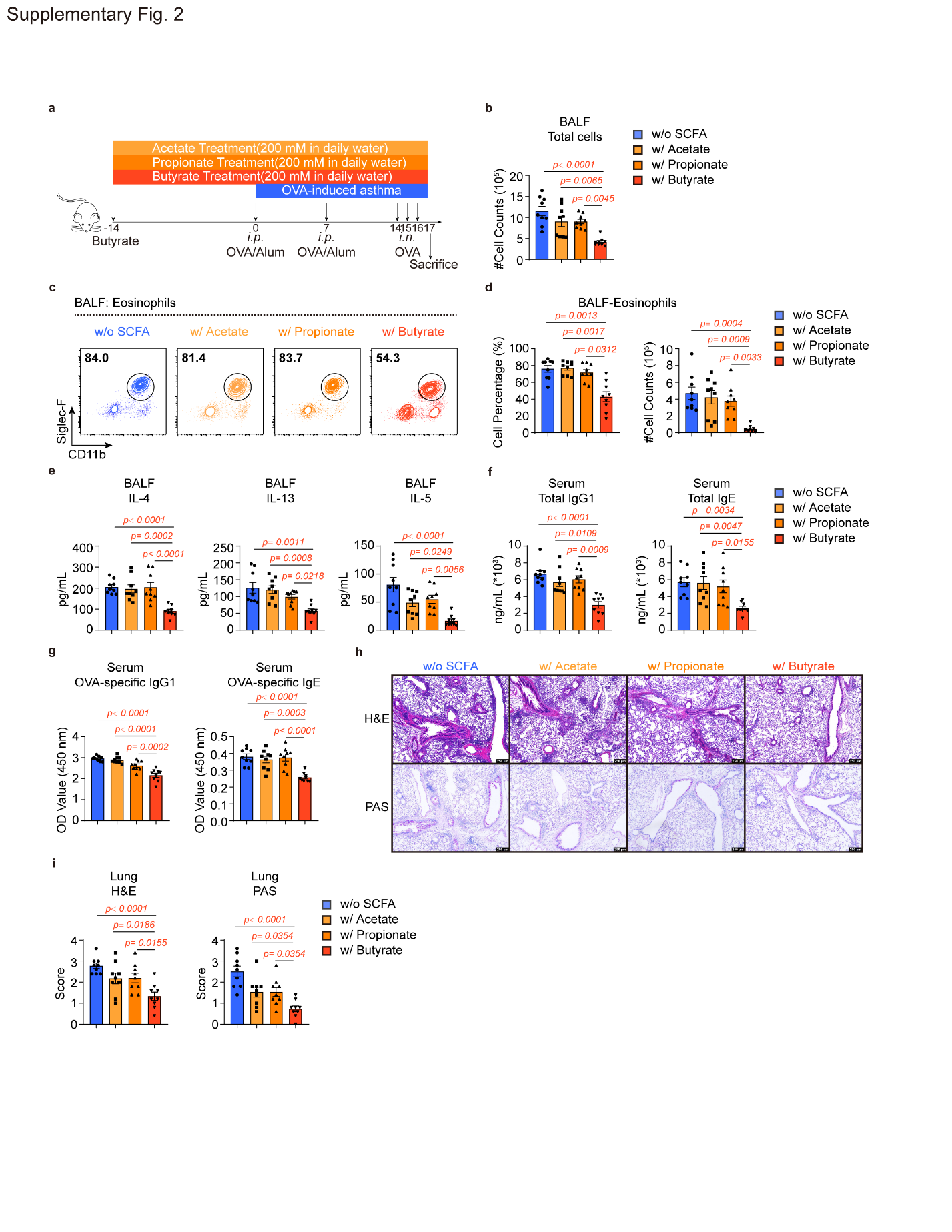


**Supplementary Fig. 2.** Butyrate, but not other SCFAs, demonstrates anti-asthmatic effects. **a** Experimental scheme. Acetate, propionate or butyrate administration was performed throughout the experimental period starting from 2 weeks prior to the induction of asthma models. **b** Total cell counts in BALF. (n=9/group) **c-d** Flow cytometry analysis of the frequencies and numbers of eosinophils in BALF. Representative plots (**c**) and statistical results (**d**) were shown. (n=9/group) **e** IL-4, IL-13, and IL-5 levels in BALF were determined by ELISA. (n=9/group) **f** Total IgG1 and IgE levels in serum. (n=9/group) **g** OVA-specific IgG1 and IgE levels in serum. (n=9/group) **h** Representative Hematoxylin and Eosin (H&E) as well as Periodic Acid-Schiff (PAS) staining of lung sections and pathological score was shown in (**i**). Scale bars represent 250 μm. (n=9/group) w/o, without; w/, with. Each symbol represents one mouse. Data combined from three independent experiments. Data represent mean ± SEM and are analyzed by one way ANOVA/nonparametric test (**b-g, i**).

**
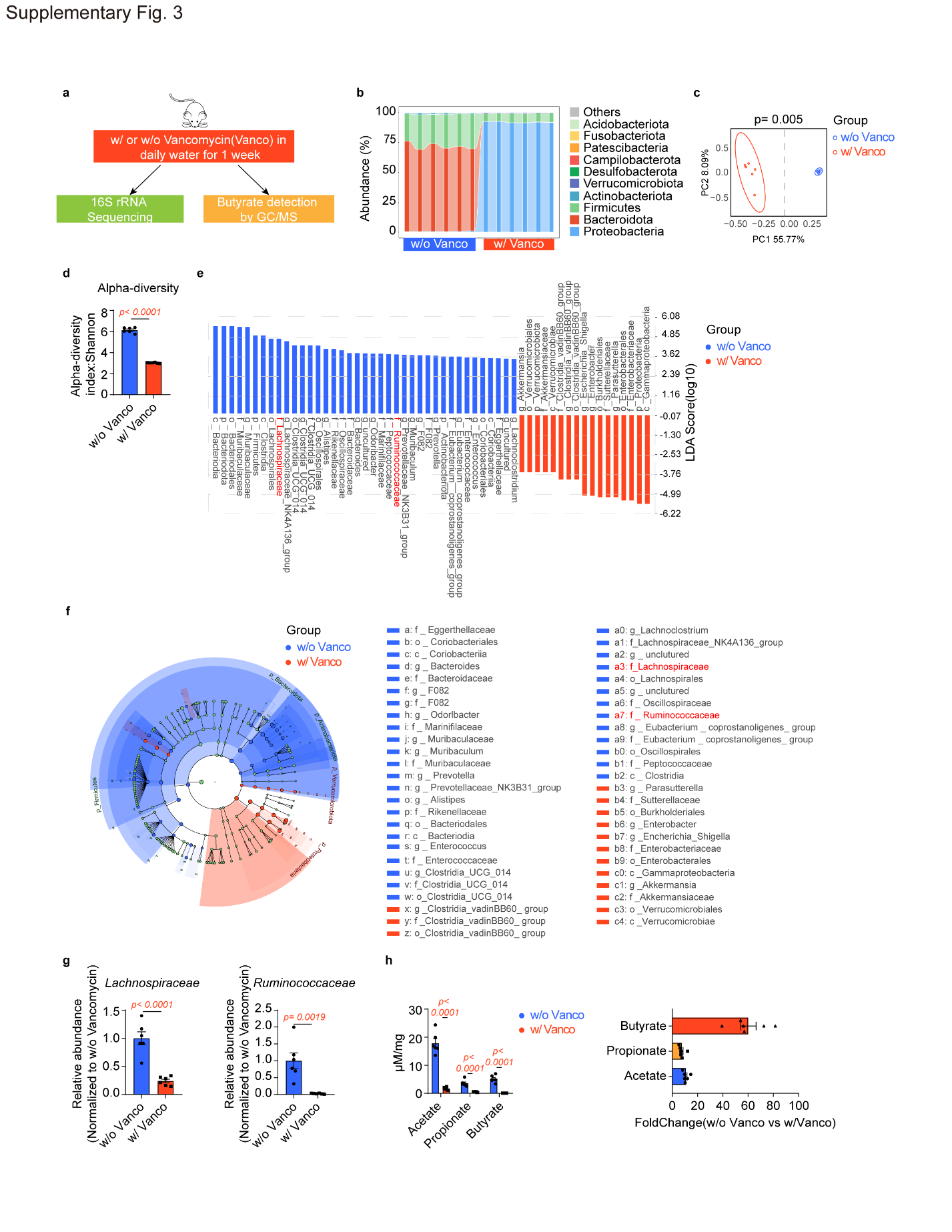
**

**Supplementary Fig. 3.** Butyrate-producing bacteria and butyrate were efficiently depleted by vancomycin treatment. **a** Experimental scheme. Mice were received water with or without vancomycin for one week. Stools and cecal contents were collected for 16S rRNA sequence and butyrate detected by GC/MS, respectively. **b** Relative abundance at phylum level of the 10 most abundant phyla in the stool of control and vancomycin-treated mice. (n=6/group) **c** Principal coordinate analysis (PcoA) of the bacterial community structure from control and vancomycin-treated mice. (n=6/group) **d** Shannon diversity index representing the alpha diversity of the bacterial community at the OTU level in the control and vancomycin-treated mice. (n=6/group) **e-f** LEfSe charts and cladogram identified the microbes whose abundances significantly differed between control and vancomycin-treated mice. (LDA score >2 & pValue<0.5) **g** qPCR for the verification of two main butyrate-producing bacteria. (n=6/group) **h** GC/MS was used to measure the levels of acetate, propionate, and butyrate in the cecal contents of mice (left panel), as well as the fold changes in different SCFAs between the two groups were shown (right panel). (n=6/group) Representative of at least two independent experiments (**g-h**). Data represent mean ± SEM and are analyzed by unpaired t test (**d,** **g-h**).


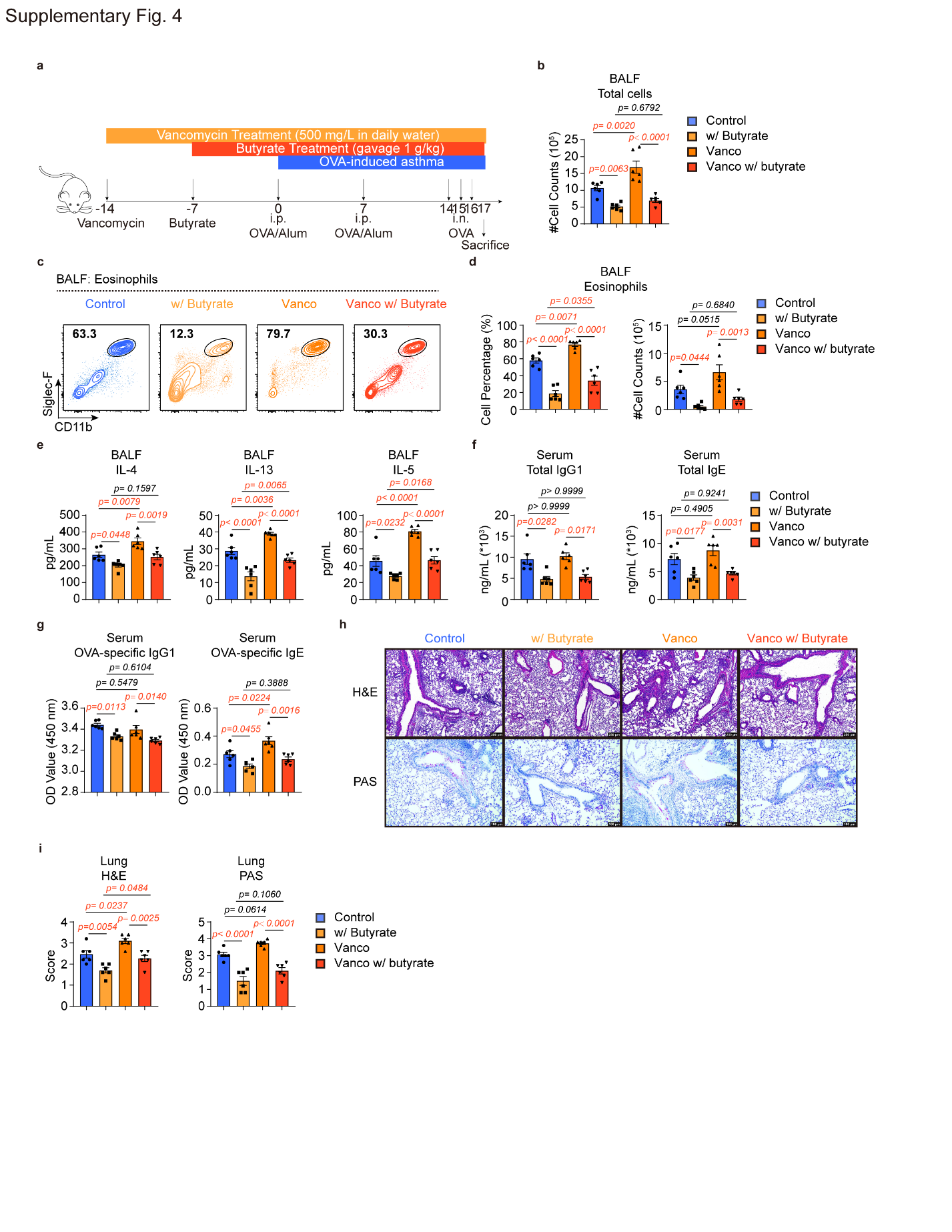


**Supplementary Fig. 4.** Depletion of endogenous butyrate exacerbates asthma progression. **a** Experimental scheme. Two weeks prior to the induction of asthma, vancomycin was utilized and throughout the experiment period. After 1 week treatment of vancomycin, mice were oral gavage daily with sodium butyrate or saline and throughout the duration of the experiment. **b** Total cell counts in BALF. (n=6/group) **c-d** Flow cytometry analysis of the frequencies of eosinophils in BALF. Representative plots (**c**) and statistical results (**d**) were shown. (n=6/group). **e** IL-4, IL-13, and IL-5 levels in BALF were determined by ELISA. (n=6/group) **f** Total IgG1 and IgE levels in serum. (n=6/group) **g** OVA-specific IgG1 and IgE levels in serum. (n=6/group) **h** Representative H&E and PAS staining of lung sections and pathological score was shown in (**i**). Scale bars represent 250 μm. (n=6/group) Each symbol represents one mouse. Representative of three independent experiments was shown and represent mean ± SEM analyzed by one-way ANOVA/nonparametric test (**b-g, i**).

**
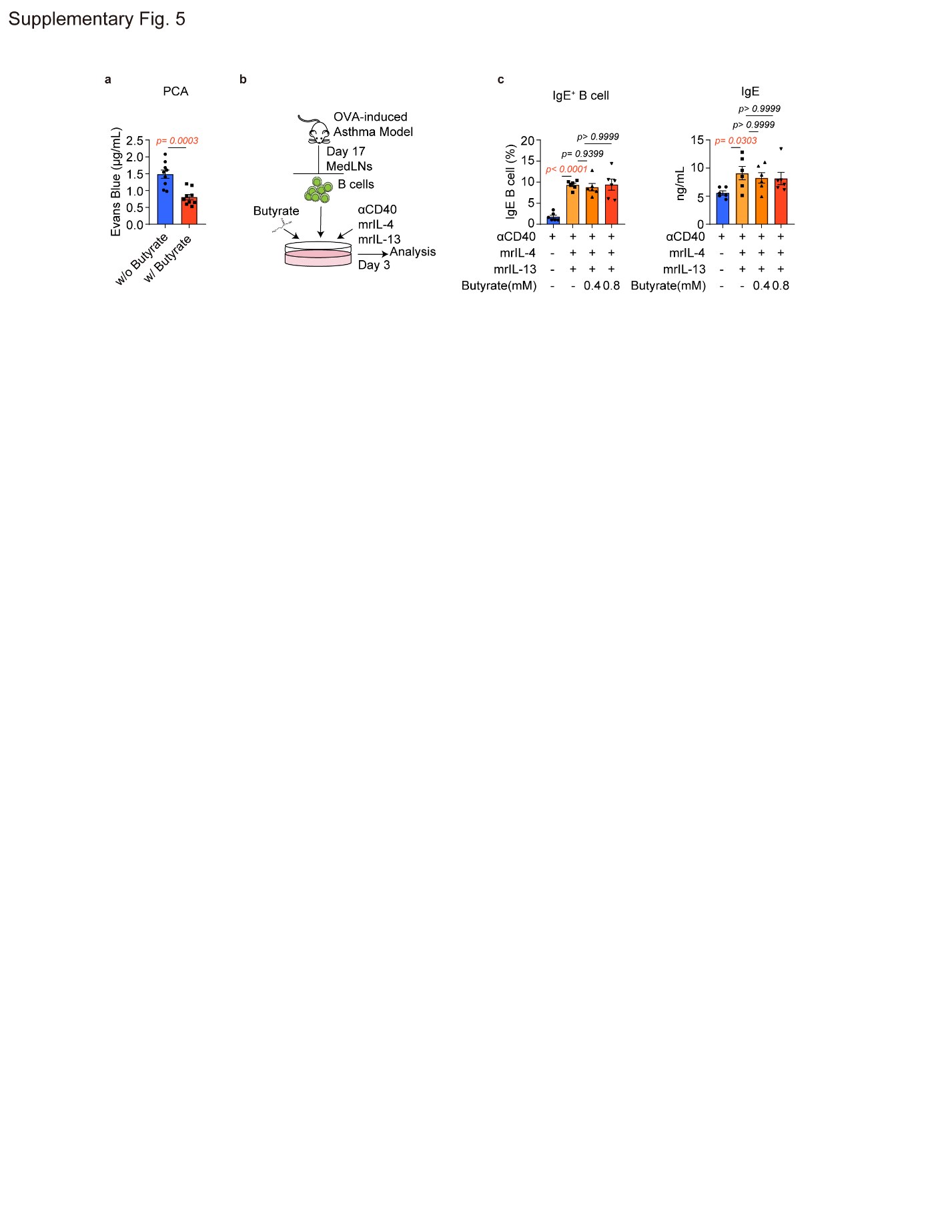
**

**Supplementary Fig. 5**. Butyrate indirectly affect IgE production. **a** Passive cutaneous anaphylaxis (PCA) assays were performed for the quantification of anaphylactic IgE using Evans Blue. (n=9/group) **b** Experiment scheme for (**c**). B cells were sorted from asthma model’s MedLNs and cultured with mrIL-4, mrIL-13 and anti-mouse CD40 in the absence or presence of butyrate (0.4 mM or 0.8 mM) for 3 days. **c** Flow cytometry and ELISA were performed for the detection of IgE^+^ B cells (left) and IgE levels (right) in the supernatant, respectively. Data were pooled from two (**c**) or three (**a**) independent experiments. Data represent mean ± SEM analyzed by unpaired t test (**a**) and one way ANOVA/nonparametric test (**c**).


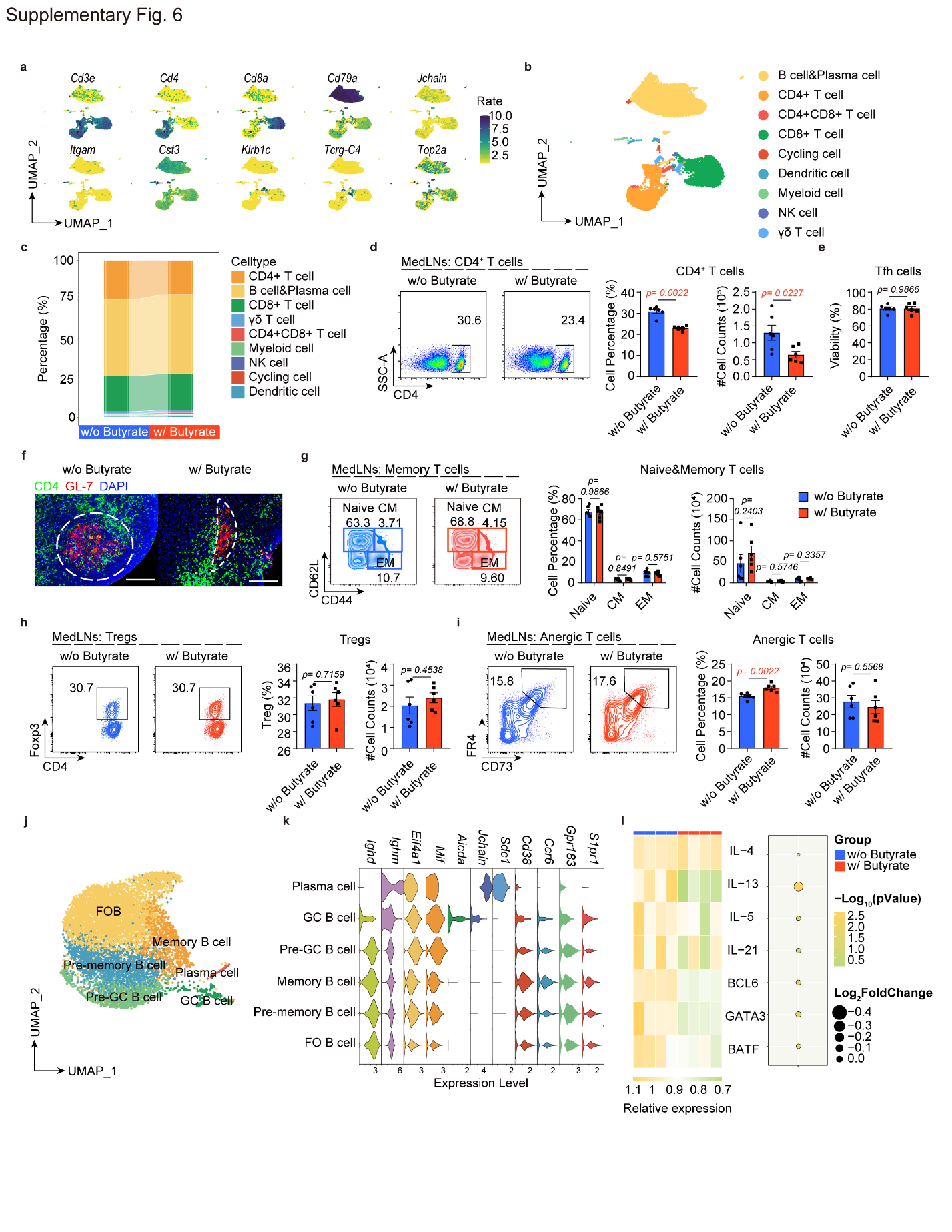


**Supplementary Fig. 6.** The effect of butyrate on different CD4^+^ T cells. **a** Selected feature genes of each cluster were shown by Uniform manifold approximation and projection (UMAP). **b** UMAP of total cell clusters. **c** Bar charts represent the proportion of each cluster in w/o Butyrate and w/ Butyrate groups. **d** Flow cytometry analysis of the frequencies and numbers of CD4^+^ T cells in MedLNs. Representative plots (left) and statistical results (right) were shown. (n=6/group) **e** Flow cytometry analysis of the viability of Tfh cells in MedLNs. Statistical results were shown. (n=6/group) **f** Representative immunofluorescent images of MedLN GCs from w/o Butyrate and w/ Butyrate groups stained for CD4 (Green), GL-7 (Red) and DAPI (Blue). Scale bars: 50 μm **g** Representative flow cytometry plots of naïve (CD62L^+^CD44^-^), central memory (CD62L^+^CD44^+^) and effector memory (CD62L^-^CD44^+^) CD4^+^ T cells (left) and statistical results (right) were shown. (n=6/group) **h** Flow cytometry analysis of the frequencies and numbers of Tregs in MedLNs. Representative plots (left) and statistical results (right) were shown. (n=6/group) **i** Flow cytometry analysis of the frequency of anergic T cells in MedLNs. Representative plots (left) and statistical results (right) were shown. (n=6/group) **j-k** UMAP of B cell clusters (**j**) and selected feature genes in each B cell cluster (**k**). **l** Flow cytometry analysis of the expression levels of key transcription factors (GATA3, BATF, BCL6) and cytokines (IL21, IL4, IL5, IL13) in Tfh cells from butyrate-treated versus control mice. The relative expression was shown. (n=4/group) Representative of three independent experiments **(d-i, l)** was shown. Data represent mean ± SEM analyzed by unpaired t test/ nonparametric test (**d-e, g-i, l**).


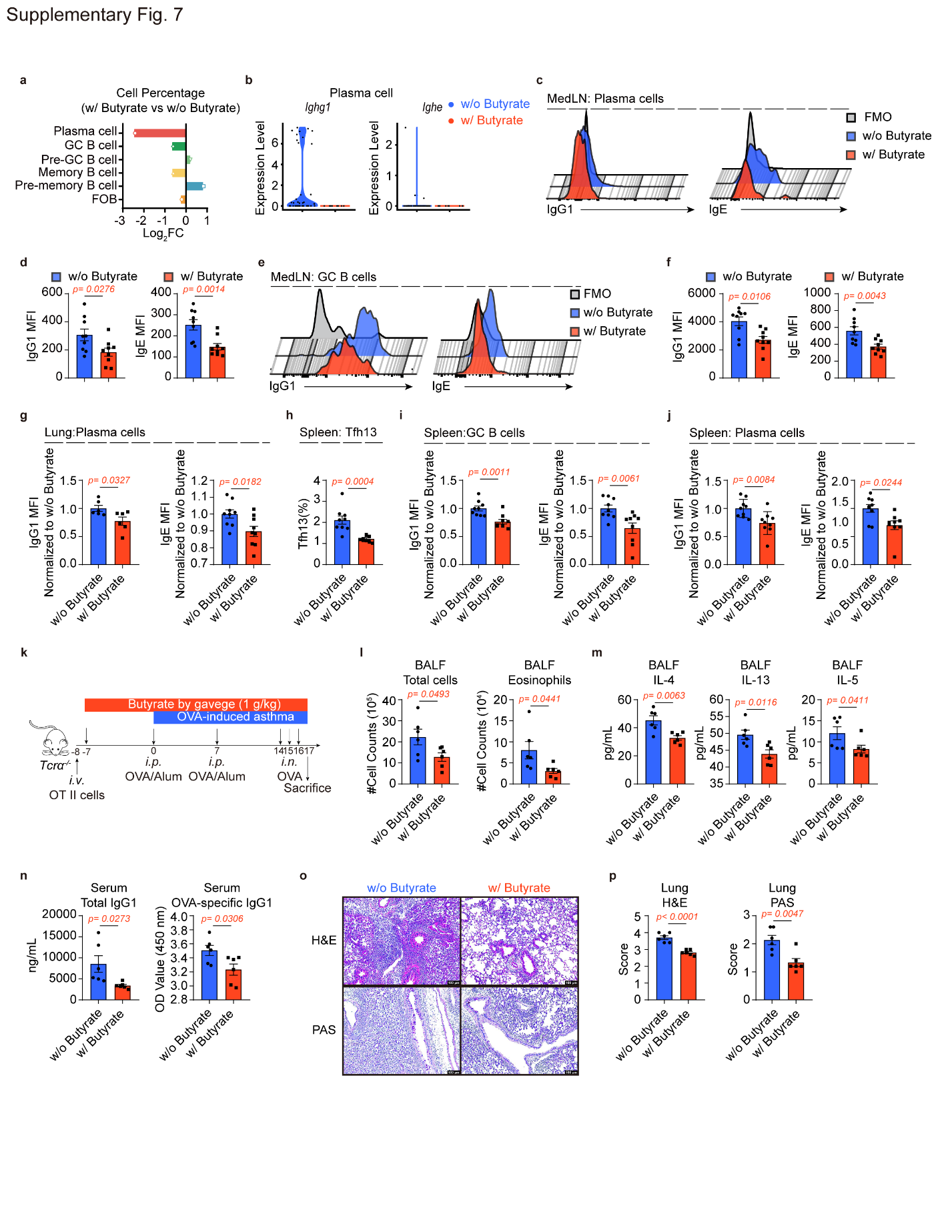


**Supplementary Fig. 7.** Impact of butyrate supplementation on B cell alterations and asthma progression in OT2 mouse model. **a** The changes in different B cell clusters (w/ Butyrate vs w/o Butyrate). **b** The *Ighg1* and *Ighe* expression levels in plasma cells of w/o Butyrate and w/ Butyrate group. **c-f** Flow cytometry analysis of the MFI of IgG1 and IgE in plasma cells and GC B cells from w/o Butyrate and w/ Butyrate group. **c** Representative histogram of IgG1 and IgE MFI in plasma cells. **d** Statistical results. (n=9/group) **e** Representative histogram of IgG and IgE MFI in GC B cells. **f** Statistical results. (n=9/group) **g** Statistical results of IgG1 and IgE MFI in the lung plasma cells from w/o Butyrate and w/ Butyrate group. (n=6-9/group) **h-j** Systemic immunity was evaluated using spleens through flow cytometry for the analysis of Tfh13, GC B and plasma cells. **h** Statistical results of the frequency of Tfh13 cells. **i** Statistical results of IgG1 and IgE MFI in GC B cells. (n=9/group) **j** Statistical results of IgG1 and IgE MFI in plasma cells. (n=9/group) **k** Experiment scheme. OT II cells were adoptively transferred into *Tcrα^-/-^* mice before butyrate administration, and then 1 week prior to the induction of asthma, butyrate by oral gavage was performed and throughout the duration of experiment. **l** Total cell and eosinophil counts in BALF. (n=6/group) **m** IL-4, IL-13 and IL-5 levels in BALF. (n=6/group) **n** Total and OVA-specific IgG1 levels were quantified using ELISA. (n=6/group) **o** Representative H&E and PAS staining of lung sections and pathological score was shown in (**p**). Scale bars represent 100 μm. (n=6/group) Data were pooled from at least two independent experiments (**d, f-j**). Representative of two independent experiments was shown in (**k-p**). Data represent mean ± SEM analyzed by unpaired t test/ nonparametric test (**d, f, g-j, l-n, p**).


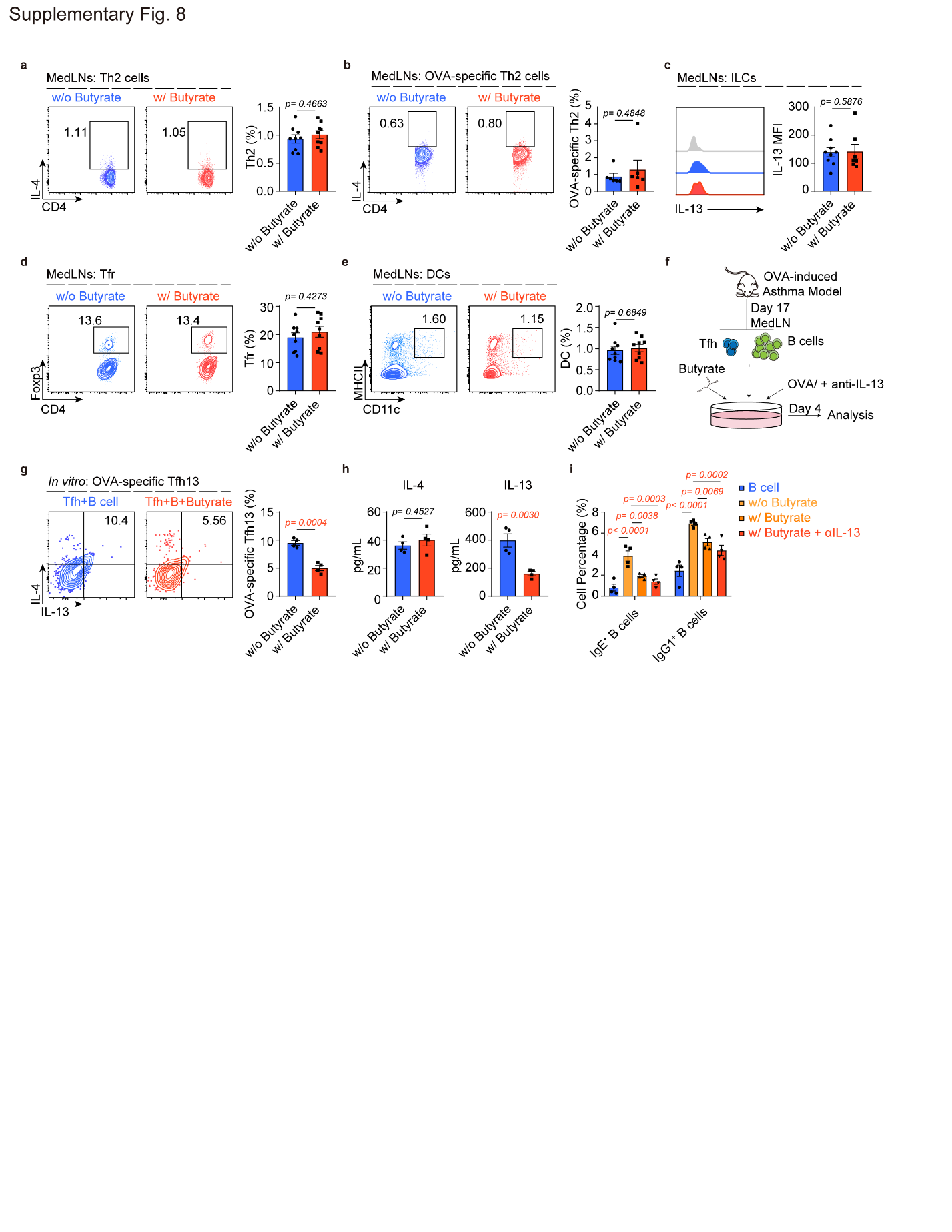


**Supplementary Fig. 8.** Changes in other immune cells in w/ Butyrate group versus w/o Butyrate group. **a** Representative flow cytometry plots of Th2 cells from w/o Butyrate and w/ Butyrate group (left) and statistical results (right) were shown. (n=9/group) **b** Representative flow cytometry plots of OVA-specific Th2 cells from w/o Butyrate and w/ Butyrate group (left) and statistical results (right) were shown. (n=6/group) **c** Representative flow cytometry histogram of IL-13 MFI in ILCs from w/o Butyrate and w/ Butyrate group (left) and statistical results (right) were shown. (n=7-9/group) **d** Representative flow cytometry plots of Tfr from w/o Butyrate and w/ Butyrate group (left) and statistical results (right) were shown. (n=9/group) **e** Representative flow cytometry plots of DC cells from w/o Butyrate and w/ Butyrate group (left) and statistical results (right) were shown. (n=9/group) **f** Experimental scheme for (**g-h**). CD4^+^CD19^-^PD-1^+^CXCR5^+^ Tfh cells and CD19^+^CD4^-^ B cells were sorted from OVA-induced asthma models and were cocultured with or without butyrate/anti-IL-13 mAb in the presence of OVA (40 μg/mL) for 4 days. **g** Representative flow cytometry plots of OVA-specific Tfh13 in w/o Butyrate and w/ Butyrate group (left) and statistical results (right) were shown. The cells were gated on Live CD4^+^I-A_b_ OVA_323-339_ Tetramer^+^ cells. **h** IL-4 and IL-13 levels in the supernatants were detected by ELISA.  **i** Statistical results of IgG1^+^ and IgE^+^ B cells in B cell group, Tfh + B group, Tfh+ B+ butyrate group as well as Tfh + B + anti-IL-13 mAb group. Each symbol represents one sample. 10-12 mice were pooled for one sample. (n=4/group) Data were pooled from at least two independent experiments (**a-e**). Representative of three independent experiments was shown in (**g-i**). Data represent mean ± SEM analyzed by unpaired t test/ nonparametric test (**a-e, g-h**) and two-way ANOVA (**i**).


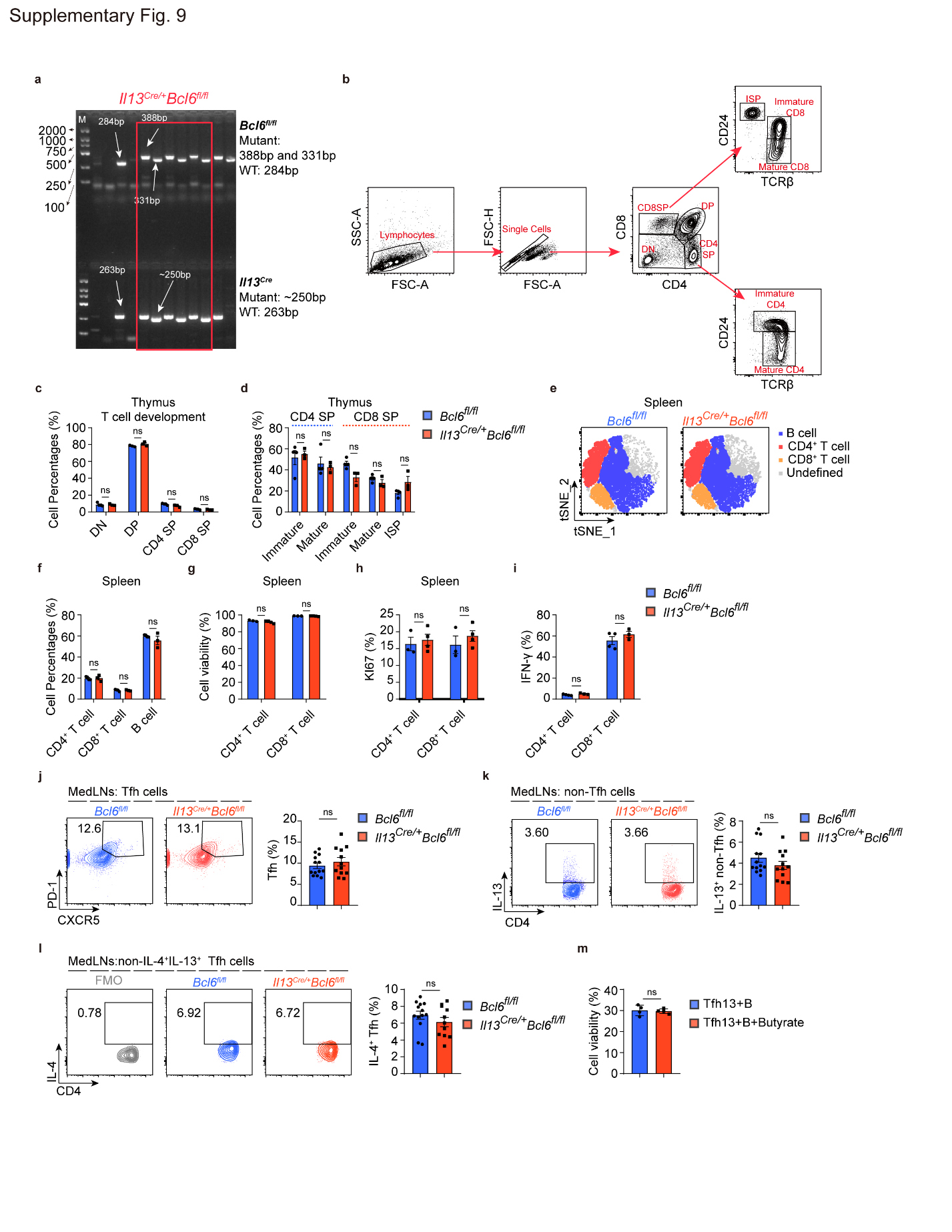


**Supplementary Fig. 9.** Tfh13 conditional knockout and its effect on T cell development and function. **a** The genotyping of *Il13^cre/+^Bcl6^fl/fl^* mice were performed using polymerase chain reaction (PCR). **b** Scheme of flow cytometry analysis of T cell development in thymus from *Bcl6^fl/fl^* and *Il13^cre/+^Bcl6^fl/fl^* mice. Statistical results were shown in (**c-d**). (n=3-4/group) **e** T-distributed Stochastic Neighbor Embedding (tSNE) of CD8^+^ T cells, CD4^+^ T cells and B cells in spleens from *Bcl6^fl/fl^* and *Il13^cre/+^Bcl6^fl/fl^* mice. Statistical results were shown in (**f**). (n=3-4/group) **g-i** T cell viability (**g**), proliferation (**h**) and function (**i**) were determined by flow cytometry and statistical results were shown. (n=3-4/group) **j** Representative flow cytometry plots of Tfh cells in the MedLNs from asthmatic *Bcl6^fl/fl^* and *Il13^cre/+^Bcl6^fl/fl^* mice (left) and statistical results (right) were shown. (n=12-13/group) **k** Representative flow cytometry plots of IL-13^+^ non-Tfh cells in MedLNs from asthmatic *Bcl6^fl/fl^* and *Il13^cre/+^Bcl6^fl/fl^* mice (left) and statistical results (right) were shown. (n=12-13/group) **l** Representative flow cytometry plots of IL-4^+^ Tfh cells in MedLNs from asthmatic *Bcl6^fl/fl^* and *Il13^cre/+^Bcl6^fl/fl^* mice (left) and statistical results (right) were shown. (n=11-13/group) **m** The cell death was determined by flow cytometry and statistical results were shown. (n=4/group) Representative of at least two independent experiments was shown in (**c-d, f-i, m**). Data pooled from three independent experiments were shown in (**j-l**). Data represent mean ± SEM analyzed by unpaired t test/ nonparametric test (**j-m**) and two-way ANOVA (**c-d, f-i**).


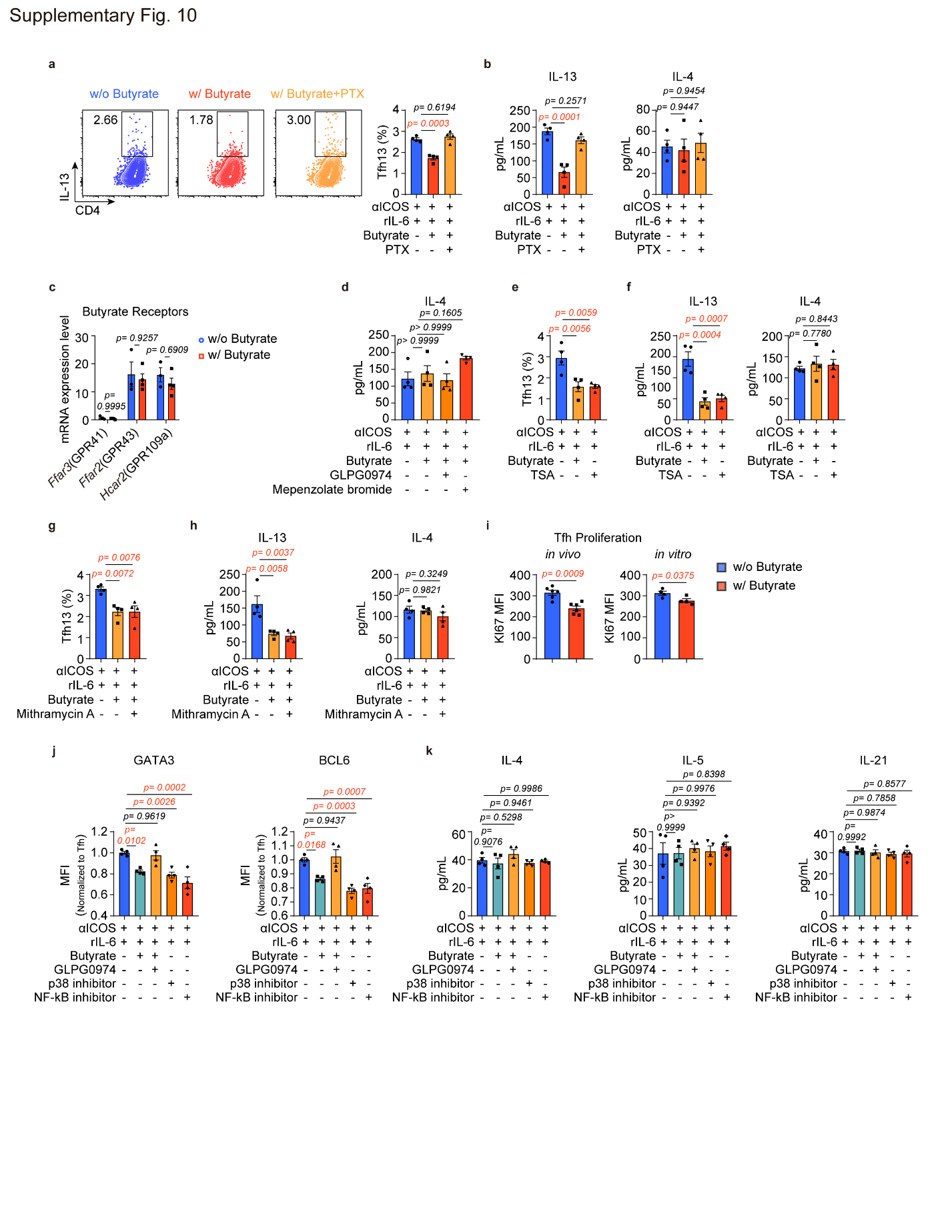


**Supplementary Fig. 10.** Butyrate inhibits Tfh13 function via GPR signaling independent of HDAC inhibition. **a-b** Tfh cells were sorted from MedLNs from asthma models and cultured *in vitro* with mrIL-6, anti-mouse ICOS mAb and anti-mouse CD3 mAb in the presence of butyrate or butyrate + Pertussis toxin (PTX). **a** Representative flow cytometry plots (left) of the frequency of Tfh13 cells. Statistical results (right) were shown. (n=4/group) **b** ELISA for IL-13 and IL-4 levels in cultured supernatant. (n=4/group) **c** qPCR for the expression of *Ffar2*, *Ffar3* and *Hcar2* mRNA levels. (n=3-4/group) **d** Tfh cells were sorted from MedLNs from asthma models and cultured *in vitro* with mrIL-6, anti-mouse ICOS mAb and anti-mouse CD3 mAb in the presence of butyrate, butyrate + GLPG0974 or butyrate + Mepenzolate bromide. ELISA for IL-4 levels in cultured supernatant. (n=4/group) **e-f** Tfh cells were sorted from MedLNs from asthma models and cultured *in vitro* with mrIL-6, anti-mouse ICOS mAb and anti-mouse CD3 mAb in the presence of butyrate or TSA. **e** Flow cytometry analysis of Tfh13 cells and statistical results were shown. (n=4/group) **f** ELISA for IL-13 and IL-4 levels in cultured supernatants. **g-h** Tfh cells were sorted from MedLNs from asthma models and cultured in vitro with mrIL-6, anti-mouse ICOS mAb and anti-mouse CD3 mAb in the presence of butyrate or butyrate + Mithramycin A. **g** Flow cytometry analysis of Tfh13 cells and statistical results were shown. (n=4/group) **h** ELISA for IL-13 and IL-4 levels in cultured supernatants. (n=4/group) **i** Flow cytometry analysis of the proliferation of the Tfh cells by assessing the expression of KI67 *in vivo* (left) and *in vitro* (right). Statistical results were shown. (n=4-6/group) **j-k** Tfh cells were sorted from MedLNs from asthma models and cultured *in vitro* with mrIL-6, anti-mouse ICOS mAb and anti-mouse CD3 mAb in the presence of butyrate, butyrate + GLPG0974, p38 MAPK inhibitor or NF-κB inhibitor.  **j** Flow cytometry analysis of GATA3 and BCL6 in cultured Tfh cells. Statistical results were shown. (n=4/group) **k** ELISA for IL-4, IL-5 and IL-21 levels in cultured supernatant. (n=4/group) Representative of at least two independent experiments was shown. Data represent mean ± SEM analyzed by one-way ANOVA/nonparametric test (**a, b, d-k**) and two-way ANOVA (**c**).

**
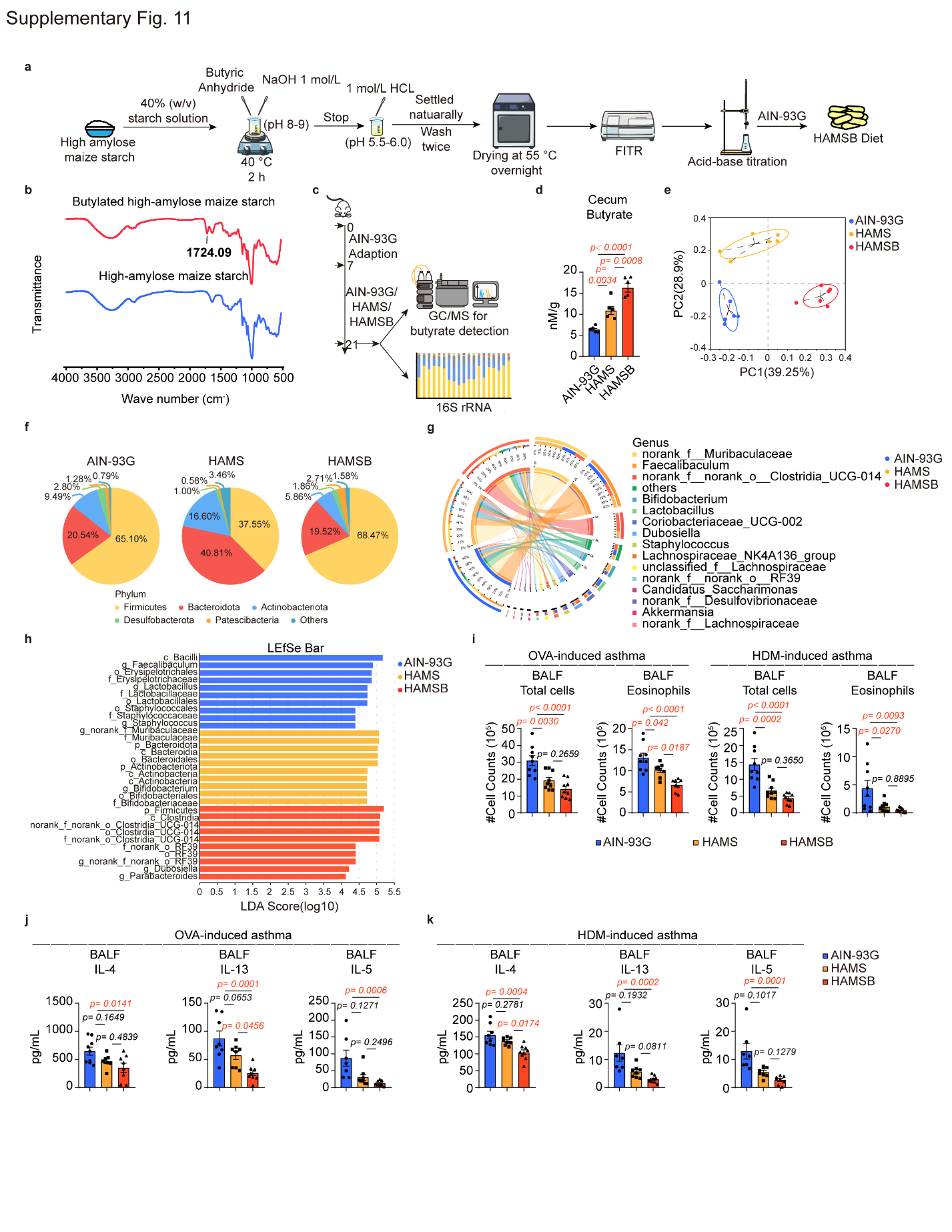
**

**Supplementary Fig. 11.** Production process of HAMSB diet and its effects on gut microbiota and airway inflammation. **a** The production process of HAMSB diet. **b** Fourier transform infrared spectrum (FTIR) spectra of high-amylose maize starch (HAMS) and butylated high-amylose maize starch (HAMSB). **c** Experimental scheme for (**d-h**). Under physiological conditions, mice were fed with AIN-93G diet (normal diet) for one week, and then randomly divided into three groups, one with AIN-93G diet, one with HAMS diet and one with HAMSB diet, for two weeks. The cecum and its contents were then collected for further microbiota and butyrate detection. (n=6/group) **d** The butyrate levels in cecum were determined by GC/MS. **e** PCoA of the bacterial community structure from AIN-93G, HAMS and HAMSB groups. **f** Pie charts represent the relative abundance of the bacterial community at the phylum level from AIN-93G, HAMS and HAMSB groups. **g** Chordal graph displays the relative abundance of the bacterial community at the genus level from AIN-93G, HAMS and HAMSB groups. **h** LEfSe charts shows the microbes whose abundances significantly differed among AIN-93G, HAMS and HAMSB groups. (Top 10 in each group) **i** Total cell and eosinophil counts in BALF of OVA-induced or HDM-induced asthma model from AIN-93G, HAMS and HAMSB groups. (n=8-9/group) **j** IL-4, IL-13 and IL-5 levels in BALF were determined by ELISA in OVA-induced asthma from AIN-93G, HAMS and HAMSB groups. (n=7-9/group) **k** IL-4, IL-13 and IL-5 levels in BALF were determined by ELISA in HDM-induced asthma from AIN-93G, HAMS and HAMSB groups. (n=7-9/group) Data were pooled from at least two independent experiments (**i-k**) or were representative of at least two independent experiments in (**b, d**). Data represent mean ± SEM analyzed by one-way ANOVA/ nonparametric test (**d, i-k**).

**
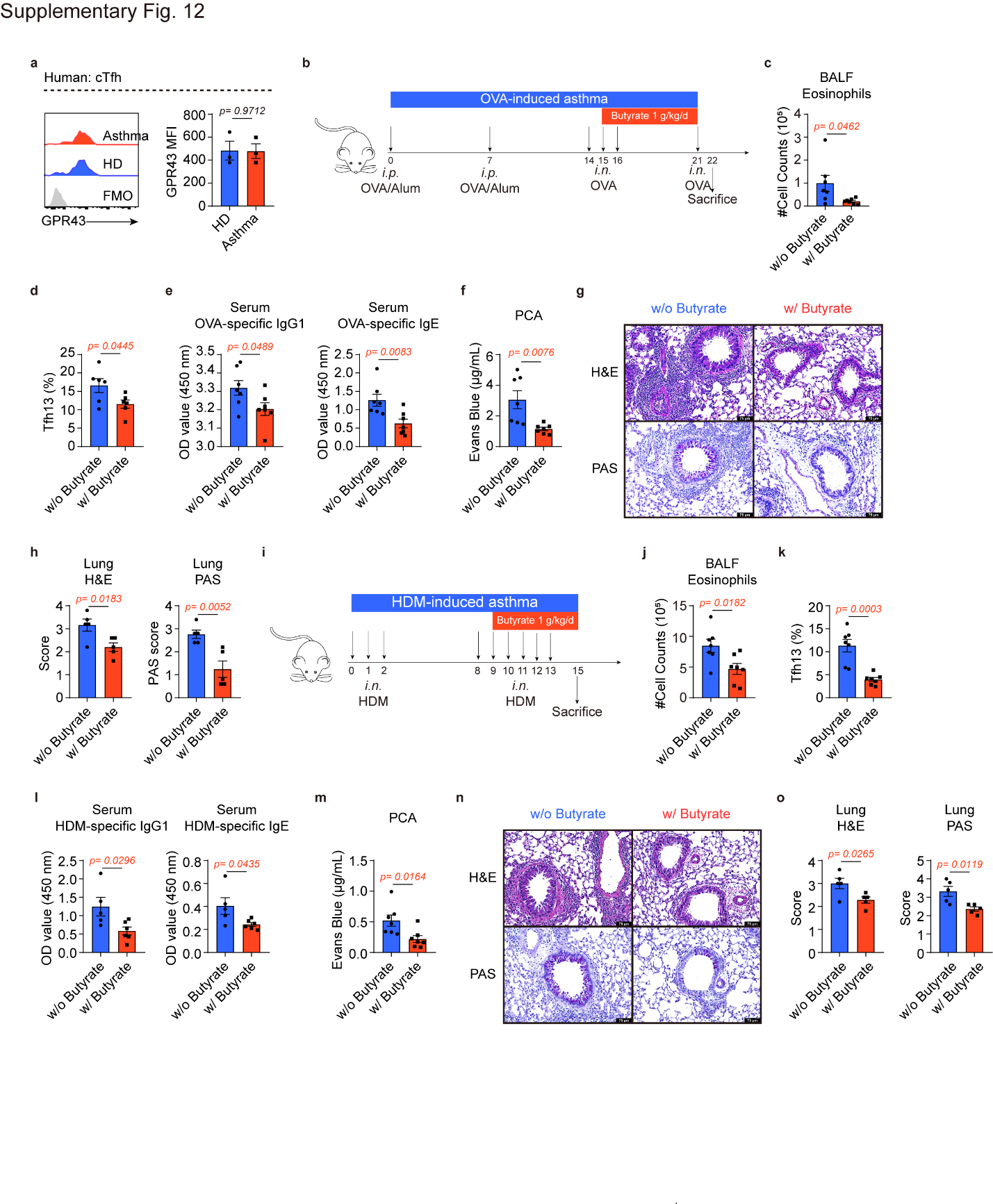
**

**Supplementary Fig. 12.** Butyrate has therapeutic effect on asthma. **a** The GPR43 expression was detected in cTfh cells from healthy donor and patients with asthma using flow cytometry. Representative histogram of GPR43 MFI (left) and statistical results (right) were shown. (n=3/group) **b** Experimental scheme. Butyrate by oral gavage 1 g/kg starts at the challenge stage of OVA-induced asthma model for 1 week. **c** Eosinophil counts in BALF. (n= 7/group) **d** Flow cytometry analysis of Tfh13 cells and statistical results were shown. (n=6/group) **e** OVA-specific IgG1 and IgE were determined by ELISA. (n=7/group) **f** Anaphylactic IgE levels were determined by PCA assays. (n= 7/group) **g** Representative H&E and PAS staining of the lung sections and pathological score was shown in (**h**). Scale bar represents 75 μm. (n= 5/group) **i** Experimental scheme. Butyrate by oral gavage 1 g/kg starts at the challenge stage of HDM-induced asthma model for 1 week. **j** Eosinophil counts in BALF. (n=7/group) **k** Flow cytometry analysis of Tfh13 cells and statistical results were shown. (n=7/group) **l** HDM-specific IgG1 and IgE levels were determined by ELISA. (n=5-6/group) **m** Anaphylactic IgE levels were determined by PCA assays. (n= 7/group) **n** Representative H&E and PAS staining of the lung sections and pathological score was shown in (**o**). Scale bar represents 75 μm. (n= 5/group) Representative of at least two independent experiments was shown. Data represent mean ± SEM analyzed by unpaired t test/ nonparametric test (**a, c-f, h, j-m, o**).

**Supplementary Tables**

**Supplementary Table 1.**

Patient demographics and clinical characteristics, related to Fig. 1b-1d, Supplementary Fig. 1a and Fig. 6a-6c.

|  | **Healthy Donor** | **Asthma Patients** |
| --- | --- | --- |
| **No.** | 25 | 25 |
| **Age (Mean)** | 52 | 55.6 |
| **Gender (male/female)** | 12/13 | 7/18 |
| **Eosinophils(10^9^/L)** | NA | 0.13±0.14 |
| **FEV1(%)** | NA | 65.7±24.64 |
| **Total IgE(IU/mL)** | NA | 178.65±124.61 |

**Supplementary Table 2.**

Patient demographics and clinical characteristics, related to Fig. 6d-6k.

|  | **Asthma Patients** |
| --- | --- |
| **No.** | 23 |
| **Age (Mean)** | 34.6 |
| **Gender (male/female)** | 11/12 |
| **Eosinophils(10^9^/L)** | 0.36±0.37 |
| **FeNO(bp)** | 65±66.73 |
| **Total IgE (IU/mL)** | 474.35±484.71 |
| **HDM-specific IgE (KUA/L)** | 26.37±27.01 |

**Supplementary Table 3.**

Characteristics of study population (National Health and Nutrition Examination Survey 2003–2018), related to Fig. 5a-5d

|  | Status | | | |  |
| --- | --- | --- | --- | --- | --- |
| Characteristic | Overall,  N = 27660 (100%)^1^ | Current asthma, N = 2327 (8.4%)^1^ | Ex-asthma,  N = 1577 (5.8%)^1^ | Non-asthma, N = 23756 (86%)^1^ | P Value^2^ |
| Sex |  |  |  |  | <0.001 |
| Female | 14194 (52%) | 1504 (65%) | 783 (51%) | 11907 (50%) |  |
| Male | 13,466 (48%) | 823 (35%) | 794 (49%) | 11,849 (50%) |  |
| Age | 46 (33, 59) | 47 (33, 59) | 40 (28, 55) | 47 (34, 59) | <0.001 |
| Race |  |  |  |  | <0.001 |
| Non-Hispanic White | 12383 (70%) | 1161 (71%) | 740 (70%) | 10482(70%) |  |
| Non-Hispanic Black | 5868 (11%) | 579 (13%) | 367 (12%) | 4922 (10%) |  |
| Mexican American | 4394 (7.9%) | 218 (4.9%) | 152 (5.2%) | 4024 (8.4%) |  |
| Other/multiracial | 2666 (7.0%) | 192 (6.8%) | 159 (7.1%) | 2315 (7.0%) |  |
| Other Hispanic | 2349 (4.8%) | 177 (3.8%) | 159 (5.7%) | 2,013 (4.9%) |  |
| Poverty Index |  |  |  |  | <0.001 |
| <1 | 5419 (13%) | 620 (20%) | 318 (15%) | 4481 (12%) |  |
| 1.1-3 | 11260 (34%) | 932 (35%) | 623 (34%) | 9705 (34%) |  |
| >3 | 10981 (52%) | 775 (45%) | 636 (51%) | 9570 (53%) |  |
| Education |  |  |  |  | <0.001 |
| Less Than 9th Grade | 2390 (4.2%) | 162 (3.5%) | 73 (2.4%) | 2155 (4.3%) |  |
| 9-11th Grade | 3651 (9.5%) | 330 (10%) | 183 (8.3%) | 3138 (9.5%) |  |
| High School Grad/GED | 6369 (23%) | 536 (23%) | 325 (20%) | 5508 (24%) |  |
| Some College or AA degree | 8455 (32%) | 815 (37%) | 590 (37%) | 7050 (31%) |  |
| College Graduate or above | 6785 (31%) | 483 (26%) | 406 (32%) | 5896 (31%) |  |
| Smoking status |  |  |  |  | 0.001 |
| Current smokers | 5784 (21%) | 603 (25%) | 347 (21%) | 4834 (20%) |  |
| Ex-smokers | 6743 (25%) | 603 (26%) | 367 (24%) | 5773 (25%) |  |
| Non smokers | 15133 (54%) | 1121 (49%) | 863 (55%) | 13149 (55%) |  |
| BMI (kg/m^2^) |  |  |  |  | <0.001 |
| Non-obese  (< 25) | 7775 (30%) | 480 (24%) | 455 (32%) | 6840 (30%) |  |
| Obese  (25 or greater) | 19885 (70%) | 1847 (76%) | 1122 (68%) | 16916 (70%) |  |
| Total calories | 2018 (1581, 2589) | 1963 (1518, 2523) | 2102 (1618, 2744) | 2018 (1586, 2584) | 0.001 |
| Total fiber | 16 (11, 22) | 15 (10, 20) | 16 (11, 22) | 16 (11, 22) | <0.001 |
| ^1^ median (IQR) for continuous; n (%) for categorical  ^2^ chi-squared test with Rao & Scott’s second-order correction; Wilcoxon rank-sum test for complex survey samples | | | | | |

**Supplementary Table 4.**

Primer sequence for real time PCR, related to Fig. 1d, Supplementary Fig. 3g, Fig. 4b and Supplementary Fig. 10c.

| Name | Primers |  | Source |
| --- | --- | --- | --- |
| *Faecalibacterium prausnitzii* | Forward | GATGGCCTCGCGTCCGATTAG | ^8^ |
|  | Reverse | CCGAAGACCTTCTTCCTCC |  |
| *Clostridium leptum* | Forward | CCGAAGACCTTCTTCCTCC | ^9^ |
|  | Reverse | ACCTTCCTCCGTTTTGTCAAC |  |
| *Clostridial cluster I* | Forward | ATGCAAGTCGAGCGAKG | ^10^ |
|  | Reverse | TATGCGGTATTAATCTYCCTTT |  |
| *Eubacterium rectale* | Forward | AAGGGAAGCAAAGCTGTG | ^11^ |
|  | Reverse | TCGGTTAGGTCACTGGCTTC |  |
| *Clostridial cluster IV* | Forward | CCTCTTGACCGGCGTGT | ^10^ |
|  | Reverse | CAGGTAGAGCTGGGCACTCTAGG |  |
| *Ruminococcus* | Forward | CTAGGTGAAGATACTGACGGTAACCTG | ^12^ |
|  | Reverse | GTATTACCGCGGCTGCTGGCAC |  |
| *Clostridial cluster XIVa* | Forward | CGGTACCTGACTAAGAAGC | ^13^ |
|  | Reverse | AGTTTYATTCTTGCGAACG |  |
| *Roseburia spp.* | Forward | GCGGTRCGGCAAGTCTGA | ^14^ |
|  | Reverse | CCTCCGACACTCTAGTMCGA |  |
| *universal bacterial genome* | Forward | ACTCCTACGGGAGGCAGC | ^15^ |
|  | Reverse | ATTACCGCGGCTGCTGGC |  |
| *Lachnospiraceae* | Forward | CCAAGGCGGCCGTACGCTGAAGCAACGCGAAGAACCTTACCA | ^15^ |
|  | Reverse | CCAAGGCGGCCGTACGCTGAAGCAACGCGAAGAACCTTACCA |  |
| *Actin* | Forward | CTACCTCATGAAGATCCTGACC | This study |
|  | Reverse | CACAGCTTCTCTTTGATGTCAC |  |
| *Il4* | Forward | TACCAGGAGCCATATCCACGGATG | This study |
|  | Reverse | TGTGGTGTTCTTCGTTGCTGTGAG |  |
| *Il13* | Forward | CTCTTGCTTGCCTTGGTGGTCTC | This study |
|  | Reverse | TTGTGTGATGTTGCTCAGCTCCTC |  |
| *Ffar2* | Forward | GCTGACAGGCTTCGGCTTCTAC | This study |
|  | Reverse | CAGAGCAGCGATCACTCCATACAG |  |
| *Ffar3* | Forward | AGTCGCCTGGTGTGGATACTGAG | This study |
|  | Reverse | GCCGAAGCAGACGAAGAAGATGAG |  |
| *Hacr2* | Forward | TGGTGGTGGCTATTGTATTCAT | This study |
|  | Reverse | ATGTAGGTAAAGCTAAGGGTGG |  |

**Supplementary Table S5**

Details of antibody used in the study.

| **Antibodies** | **Source** | **Clone** | **Dilution** |
| --- | --- | --- | --- |
| APC anti-mouse CD19 | Biolegend | 6D5 | 1:400 |
| PE/Cyanine7 anti-mouse CD19 | Biolegend | 6D5 | 1:400 |
| Brilliant Violet 510™ anti-mouse CD19 | Biolegend | 6D5 | 1:400 |
| FITC anti-mouse CD19 | Biolegend | 6D5 | 1:400 |
| FITC anti-mouse IgE | Biolegend | RME-1 | 1:100 |
| PE anti-mouse IgE | Biolegend | RME-1 | 1:200 |
| eBioscience™ Fixable Viability Dye eFluor™ 506 | Invitrogen |  | 1:1000 |
| PE/Cyanine7 anti-mouse CD11c | Biolegend | N418 | 1:300 |
| V450 Rat anti-mouse CD11b | BD Bioscience | M1/70 | 1:400 |
| PE Rat anti-mouse Siglec-F | BD Bioscience | E50-2440 | 1:300 |
| PerCP/Cyanine5.5 anti-mouse TCR β chain | Biolegend | H57-597 | 1:300 |
| FITC anti-Human/Mouse CD44 | Biolegend | IM7 | 1:600 |
| APC/Fire™ 750 anti-mouse CD4 | Biolegend | RM4-5 | 1:400 |
| Alexa Fluor® 700 anti-mouse CD4 | Biolegend | GK1.5 | 1:400 |
| PE anti-mouse CD4 | Biolegend | RM4-5 | 1:400 |
| CD4 Monoclonal Antibody (RM4-5), PE-Cyanine7, eBioscience™ | Invitrogen | RM4-5 | 1:400 |
| Anti-CD4 antibody [EPR6855] | Abcam |  | 1:200(IF) |
| APC anti-mouse CD279(PD-1) | Biolegend | RMPI-30 | 1:200 |
| Alexa Fluor® 647 anti-mouse CD279 (PD-1) | Biolegend | 29F.1A12 | 1:200 |
| PE anti-mouse CD279(PD-1) | Biolegend | RMPI-30 | 1:200 |
| APC/Fire™ 750 anti-mouse CD279(PD-1) | Biolegend | 29F.1A12 | 1:200 |
| CD185 (CXCR5) Monoclonal Antibody (SPRCL5), APC, eBioscience™ | Invitrogen | SPRCL5 | 1:200 |
| Brilliant Violet 421™ anti-mouse CD185 (CXCR5) | Biolegend | L138D7 | 1:200 |
| FITC anti-mouse I-A/I-E | Biolegend | M5/114.15.2 | 1:400 |
| PE anti-mouse IL-4 | Biolegend | 11B11 | 1:100 |
| PE-Cy™7 Rat Anti-Mouse IL-4 | BD Bioscience | 11B11 | 1:100 |
| IL-4 Monoclonal Antibody (11B11), APC, eBioscience™ | Invitrogen | 11B11 | 1:100 |
| IL-13 Monoclonal Antibody (eBio13A), PE-Cyanine7, eBioscience™ | Invitrogen | eBio13A | 1:100 |
| IL-13 Monoclonal Antibody (eBio13A), PE, eBioscience™ | Invitrogen | eBio13A | 1:100 |
| FITC anti-mouse/human GL7 Antigen (T and B cell Activation Marker) | Biolegend | GL7 | 1:600 |
| PE anti-mouse/human GL7 Antigen (T and B cell Activation Marker) | Biolegend | GL7 | 1:600 |
| GL7 Monoclonal Antibody (GL-7 (GL7)), eBioscience™ | Invitrogen | GL7 | 1:100(IF) |
| Brilliant Violet 510™ anti-mouse CD138 (Syndecan-1) | Biolegend | 281-2 | 1:500 |
| Alexa Fluor® 647 anti-mouse/rat/human FOXP3 Antibody | Biolegend | 150D | 1:50 |
| Brilliant Violet 421™ anti-mouse CD45 | Biolegend | 30-F11 | 1:400 |
| Pacific Blue™ anti-mouse CD45 | Biolegend | 30-F11 | 1:200 |
| eBioscience™ Fixable Viability Dye eFluor™ 660 | Invitrogen |  | 1:1000 |
| PE/Cyanine7 anti-mouse CD62L | Invitrogen | MEL-14 | 1:400 |
| PE/Cyanine7 anti-mouse IgG1 | Biolegend | RMG1-1 | 1:500 |
| APC anti-human CD4 | Biolegend | OKT4 | 1:50 |
| APC/Fire™ 750 anti-human CD185(CXCR5) | Biolegend | J252D4 | 1:20 |
| Brilliant Violet 421™ anti-human CD45RA | Biolegend | HI100 | 1:100 |
| PE anti-human IL-13 | Biolegend | JES10-5A2 | 1:20 |
| PE/Cyanine7 anti-human IL-4 | Biolegend | MP4-25D2 | 1:20 |
| ABflo®647 Rabbit anti-mouse CD95/FAS | ABclonal | ARC61283 | 1:200 |
| FITC anti-mouse TCR Vα2 | Biolegend | B20.1 | 1:300 |
| PerCP/Cyanine5.5 anti-mouse/human CD44 | Biolegend | IM7 | 1:600 |
| PE anti-mouse/human Ki-67 | Biolegend | 11F6 | 1:50 |
| Goat anti-mouse IgG1-HRP | SouthernBiotech |  | 1:4000(ELISA) |
| Goat anti-mouse IgE-HRP | SouthernBiotech |  | 1:2000(ELISA) |
| APC anti-mouse CD24 | Biolegend | M1/69 | 1:400 |
| V450 anti-mouse IFNγ | Invitrogen | XMG1.2 | 1:200 |
| PE anti-mouse BATF | BD Bioscience | S39-1060 | 1:50 |
| PE/Cyanine7 anti-mouse/human GATA3 | Invitrogen | TWAJ | 1:50 |
| APC anti-mouse CD73 | Biolegend | TY/11.8 | 1:200 |
| PE anti-mouse FR4 | Biolegend | TH6 | 1:200 |
| GPR43 antibody | MCE |  | 1:100 |
| Goat anti-Rabbit IgG (H+L) Cross-Adsorbed Secondary Antibody, Alexa Fluor™ 488 | Invitrogen |  | 1:100 |
| Purified anti-mouse CD16/32 | Biolegend | 93 | 1:100 |
| Brilliant Violet 421™ anti-mouse Lineage Cocktail | Biolegend | 17A2; RB6-8C5; RA3-6B2; Ter-119; M1/70; | 1：50 |
| Phospho-p38 MAPK (Thr180, Tyr182) Monoclonal Antibody, APC | Invitrogen | 4NIT4KK | 1:100 |
| Rabbit anti-mouse NF-kB p65 | CST | D14E12 | 1:1000 |
| Rabbit anti-mouse p38 MAPK | Abclonal | ARC0201 | 1:1000 |
| Phospho-NFkB p65 (Ser536) Monoclonal Antibody | Invitrogen | T.849.2 | 1:1000(Immunoblot)  1:100 |
| Rabbit anti-mouse phopho-p38 MAPK | CST | 12FB | 1:1000 |
| GAPDH, Rabbit pAb | YEASEN |  | 1:5000 |
| Anti-rabbit IgG, HRP-linked antibody | CST |  | 1:5000 |
| I-A_b_ OVA_323-339_ Tetramer-APC | MBL |  | 1:5 |
| Goat anti-Rabbit IgG (H+L) Cross-Adsorbed Secondary Antibody, Alexa Fluor™ 594 | Invitrogen |  | 1:100 |

**References**

1 Slovin, S. *et al.* Single-Cell RNA Sequencing Analysis: A Step-by-Step Overview. *Methods Mol Biol* **2284**, 343-365 (2021).

2 Jin, S. *et al.* Inference and analysis of cell-cell communication using CellChat. *Nat Commun* **12**, 1088 (2021).

3 Curtin, L. R. *et al.* National Health and Nutrition Examination Survey: sample design, 2007-2010. *Vital Health Stat 2*, 1-23 (2013).

4 Zhang, H. *et al.* Reactive oxygen species stimulated pulmonary epithelial cells mediate the alveolar recruitment of FasL(+) killer B cells in LPS-induced acute lung injuries. *J Leukoc Biol* **104**, 1187-1198 (2018).

5 Gowthaman, U. *et al.* Identification of a T follicular helper cell subset that drives anaphylactic IgE. *Science* **365** (2019).

6 Hanashiro, J. *et al.* Schizophyllum commune induces IL-17-mediated neutrophilic airway inflammation in OVA-induced asthma model mice. *Sci Rep* **9**, 19321 (2019).

7 He, Y. *et al.* Butylated starch alleviates polycystic ovary syndrome by stimulating the secretion of peptide tyrosine-tyrosine and regulating faecal microbiota. *Carbohydr Polym* **287**, 119304 (2022).

8 Wang, R. F., Cao, W. W. & Cerniglia, C. E. PCR detection and quantitation of predominant anaerobic bacteria in human and animal fecal samples. *Appl Environ Microbiol* **62**, 1242-1247 (1996).

9 Lay, C. *et al.* Design and validation of 16S rRNA probes to enumerate members of the Clostridium leptum subgroup in human faecal microbiota. *Environ Microbiol* **7**, 933-946 (2005).

10 Song, Y., Liu, C. & Finegold, S. M. Real-time PCR quantitation of clostridia in feces of autistic children. *Appl Environ Microbiol* **70**, 6459-6465 (2004).

11 Balamurugan, R. *et al.* Molecular studies of fecal anaerobic commensal bacteria in acute diarrhea in children. *J Pediatr Gastroenterol Nutr* **46**, 514-519 (2008).

12 Shimokawa, C. *et al.* CD8(+) regulatory T cells are critical in prevention of autoimmune-mediated diabetes. *Nat Commun* **11**, 1922 (2020).

13 Matsuki, T. *et al.* Development of 16S rRNA-gene-targeted group-specific primers for the detection and identification of predominant bacteria in human feces. *Appl Environ Microbiol* **68**, 5445-5451 (2002).

14 Walker, A. W., Duncan, S. H., McWilliam Leitch, E. C., Child, M. W. & Flint, H. J. pH and peptide supply can radically alter bacterial populations and short-chain fatty acid ratios within microbial communities from the human colon. *Appl Environ Microbiol* **71**, 3692-3700 (2005).

15 Yang, K. *et al.* Suppression of local type I interferon by gut microbiota-derived butyrate impairs antitumor effects of ionizing radiation. *J Exp Med* **218** (2021).
