# Supplementary figures and images for: Microbiota-derived butyrate alleviates asthma via inhibiting Tfh13-mediated IgE production
Source: Signal Transduct Target Ther. 2025 Jun 6;10:181. doi: 10.1038/s41392-025-02263-2 (PMC12141656; doi:10.1038/s41392-025-02263-2)

Supplementary Fig. 9a

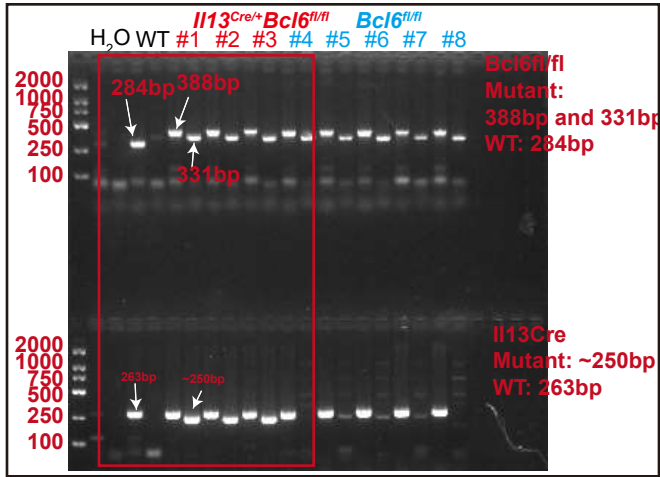

Fig.4I

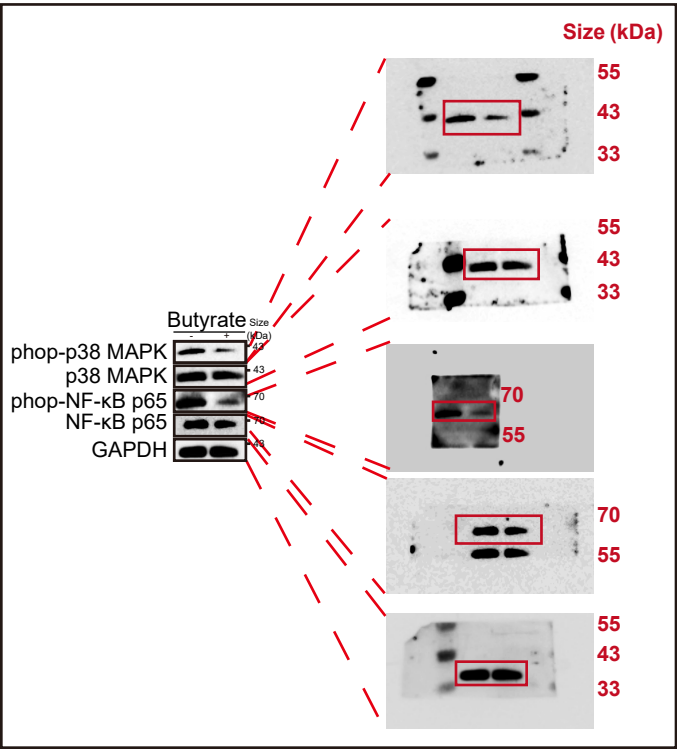

Supplement: Supplementary file 2 — Uncropped gels for genotyping and immunoblot [file 41392_2025_2263_MOESM2_ESM.pdf]
